# Transforming a head direction signal into a goal-oriented steering command

**DOI:** 10.1038/s41586-024-07039-2

**Published:** 2024-02-07

**Authors:** Elena A. Westeinde, Emily Kellogg, Paul M. Dawson, Jenny Lu, Lydia Hamburg, Benjamin Midler, Shaul Druckmann, Rachel I. Wilson

**Affiliations:** 1 Department of Neurobiology, Harvard Medical School, Boston, MA USA; 2 Department of Neurobiology, Stanford University School of Medicine, Stanford, CA USA; 3 Wu Tsai Neurosciences Institute, Stanford University, Stanford, CA USA

**Keywords:** Network models, Navigation

## Abstract

To navigate, we must continuously estimate the direction we are headed in, and we must correct deviations from our goal^1^. Direction estimation is accomplished by ring attractor networks in the head direction system^2,3^. However, we do not fully understand how the sense of direction is used to guide action. *Drosophila* connectome analyses^4,5^ reveal three cell populations (PFL3R, PFL3L and PFL2) that connect the head direction system to the locomotor system. Here we use imaging, electrophysiology and chemogenetic stimulation during navigation to show how these populations function. Each population receives a shifted copy of the head direction vector, such that their three reference frames are shifted approximately 120° relative to each other. Each cell type then compares its own head direction vector with a common goal vector; specifically, it evaluates the congruence of these vectors via a nonlinear transformation. The output of all three cell populations is then combined to generate locomotor commands. PFL3R cells are recruited when the fly is oriented to the left of its goal, and their activity drives rightward turning; the reverse is true for PFL3L. Meanwhile, PFL2 cells increase steering speed, and are recruited when the fly is oriented far from its goal. PFL2 cells adaptively increase the strength of steering as directional error increases, effectively managing the tradeoff between speed and accuracy. Together, our results show how a map of space in the brain can be combined with an internal goal to generate action commands, via a transformation from world-centric coordinates to body-centric coordinates.

## Abstract (web summary)

Here we show how PFL2 and PFL3 neurons in the *Drosophila* brain compare a representation of direction with internal spatial goals, both anchored in world-centric coordinates, and produce body-centric steering commands that act to correct deviations from the goal direction.

## Main

**Fig. 1:**
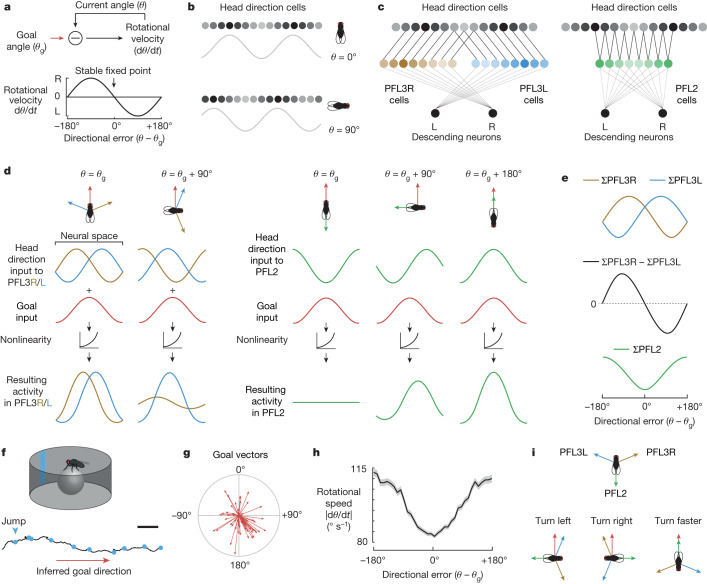
Comparing model predictions with behaviour. **a**, A rotational servomechanism works to keep the angle *θ* of some device close to a goal value *θ*_g_. The output is a rotational velocity command that depends on the system’s error (*θ* − *θ*_g_). Rotational velocity is close to zero around the goal (*θ* = *θ*_g_) and the anti-goal (*θ* = *θ*_g_ + 180°). Whereas the goal is a stable fixed point, the anti-goal is an unstable fixed point. **b**, In the *Drosophila* brain, head direction is represented in Δ7 cells as a sinusoid over two spatial cycles. **c**, PFL3L, PFL3R and PFL2 populations extract spatially shifted copies of the head direction representation. These three populations are aligned in the fan-shaped body, where they share inputs from putative goal cells (Extended Data Fig. 1c). **d**, Model: each PFL population adds its head direction input with a shared input from goal cells. This is passed through a nonlinearity and then integrated over space. **e**, Model: activity of each PFL population versus directional error. **f**, Data: path of a fly in a virtual environment with a visual head direction cue (a bright bar). Dots indicate 90° and 180° jumps of the environment; here the fly is correcting for all these jumps with rapid turns. **g**, Mean head direction *θ* in 10 min epochs with periodic jumps. Radial length denotes the consistency of head direction over time *ρ*, which ranges here from 0 to 0.8 in *n* = 56 epochs from 56 flies; 0° is towards the cue. **h**, Data: mean rotational speed versus directional error, the s.e.m. across flies (*n* = 46 flies). **i**, Model: PFL populations have shifted head direction inputs that tile the space of compass directions. Each population detects overlap between its shifted head direction vectors and a shared goal vector. The PFL3L population drives left turning, whereas the PFL3R population drives right turning and PFL2 drives increased rotational speed. Scale bar (**f**), 30 mm.

Accurate navigation requires us to fix a goal direction and then maintain our orientation towards that goal in the face of perturbations. This is also a basic problem in mechanical engineering: how can we keep the angle of some device directed at a target^6^? One solution to this problem is to use a resolver servomechanism to measure the discrepancy or error between the current angle and the goal angle. This produces a rotational velocity command that varies sinusoidally with error (Fig. 1a). Specifically, the mechanism drives leftward rotation when the device is positioned to the right of the goal, and vice versa. The stable fixed point of this system is the angle where the rotational velocity command crosses zero with negative slope (Fig. 1a).

**Extended Data Fig. 1:**
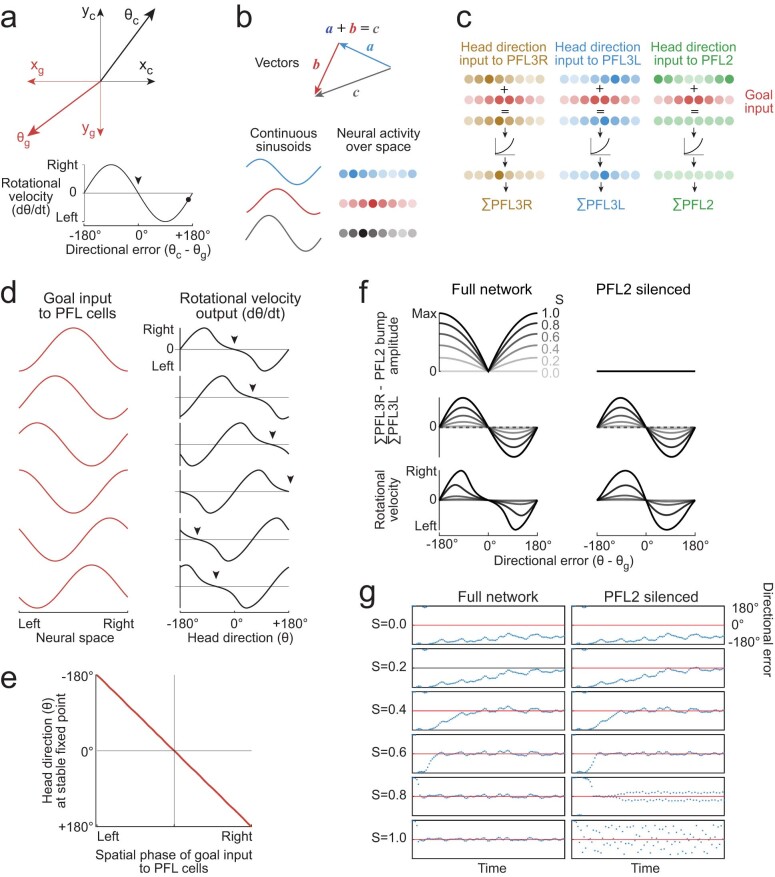
Model predictions. **a**, A resolver measures the current angle of some object ($\theta$_c_, e.g., the angular position of a shaft) and resolves that angle into its Cartesian components, x_c_ and y_c_. The goal angle $\theta$_g_ is similarly resolved into its Cartesian components, x_g_ and y_g_. These components are cross-multiplied, and their difference is used to generate a rotational velocity command *dθ*/*d*t ∝ x_c_y_g_ - x_g_y_c_. We treat positive velocity values as clockwise (CW) rotations. In this example, the current angle is rotated CW relative to the goal, meaning a positive directional error. This drives a CCW rotation. But because the error at this point (●) is almost 180°, rotational speed will be small. Mittelstaedt suggested that a similar process might be implemented in the brain’s navigation centres to control an organism’s heading, and thus its path through the environment; this is known as “Mittelstaedt ‘s bicomponent model” of steering control^7^. Arrowhead denotes the system’s stable fixed point. **b**, Any vector can be represented as a sinusoidal function whose amplitude represents the magnitude of the vector, and whose phase represents the angle of the vector. Although it is convenient to represent these sinusoids as continuous functions, they can also be discretized into spatial activity patterns over neural space^15,16^. Adding these sinusoids is equivalent to performing vector addition. **c**, Model elements shown in Fig. 1d, here schematized as spatial activity patterns over neural space. The horizontal axis of this space represents the horizontal axis of the fan-shaped body. d, Model: goal input to PFL2&3 cells (left). When this spatial pattern is shifted leftward, this produces a clockwise shift in the model’s rotational velocity as a function of head direction (right). Arrowheads denote the system’s stable fixed point. **e**, Model: shifts in the spatial phase of goal input to PFL cells produce equal shifts in the head direction corresponding to the system’s stable fixed point. This is true for all values of *S* > 0. **f**, Model: The effect of silencing PFL2 cells on rotational velocity is similar to the effect of removing the indirect pathway (compare with Fig. 5c). In both cases, the rotational velocity function becomes equally steep around the goal and the anti-goal. **g**, Model: The effect of silencing PFL2 cells on steering dynamics is similar to the effect of removing the indirect pathway (compare with Fig. 5d). In both cases, the system oscillates when *S* is high.

Sixty years ago, Mittelstaedt suggested that a similar process might be implemented in the brain’s navigation centres to control an organism’s heading and thus its path through the environment^7^. Since then, Webb and colleagues have proposed neural network implementations of this idea^8–11^, which have been extended by other investigators^4,5,12–14^. All these models exploit the notion that an angle or vector can be represented as a sinusoidal spatial pattern of activity across a neural population^15,16^ (Extended Data Fig. 1). These sinusoids can then be combined to produce a directional control signal^9^.

Data from locusts^17^, zebrafish^18^ and *Drosophila*^19^ show that head direction is in fact encoded as a sinusoidal spatial pattern of activity (Fig. 1b). The *Drosophila* brain contains a cell type (PFL3) that is anatomically positioned to receive shifted copies of this head direction representation while also making direct lateralized connections onto descending neurons involved in steering^4,5^ (Fig. 1c). This ‘copy-and-shift’ architecture^9,20^ is reminiscent of the design of a resolver servomotor (Extended Data Fig. 1). PFL3 cells also receive anatomical input from the fan-shaped body, a brain region where goals might be stored (Fig. 1d). Notably, almost all the inputs to PFL3 cells are shared by another cell type, PFL2 (refs. ^5,21^). Individual PFL2 cells make bilateral connections onto descending neurons (Fig. 1c), implying that they do not guide steering. Their function is enigmatic, but proposals suggest they increase forward walking speed^5,13^.

In short, both PFL2 and PFL3 cells are anatomically positioned to integrate head direction information with stored goal information for navigation control. These cells stand out because they form a link between an allocentric map of space and an egocentric system of motor control. Encouragingly, recordings from analogous cells in other insects have confirmed that they receive topographic input from the head direction system^17,22,23^. However, there have been no functional studies of these cells in *Drosophila*, and recent models have made conflicting predictions about their roles in motor control^4,5,11–13^.

## Comparing model predictions with behaviour

To begin, we describe an updated computational model that differs from previous models in several key ways (Methods). In this model, direction is represented as a sinusoid^24^ whose phase rotates as direction changes, relative to a flexible and arbitrary offset^19^. We divide PFL3 cells into two populations (PFL3R and PFL3L) that converge onto right or left descending neurons, respectively (Fig. 1c). Each population extracts a copy of the head direction representation, with phase shifts of ±67.5°, relative to the original head direction representation. Meanwhile, PFL2 cells extract a head direction representation with a phase shift of 180° (Fig. 1c).

These three PFL populations are aligned within the fan-shaped body, where they share inputs from orderly arrays of cells^5^ which could represent the goal angle, $\theta$_g_. We model the goal representation as a spatial sinusoid whose phase represents $\theta$_g_ (Fig. 1d). The firing rate of each model PFL cell is the sum of its head direction input and its goal input, passed through a nonlinearity (Fig. 1d).

These sinusoids should be understood as representations of vectors (Extended Data Fig. 1): the two PFL3 populations extract shifted copies of the head direction vector, and the goal vector is added to each copy. The resulting vector with the larger magnitude dictates the direction the fly should rotate to reach its goal. This model predicts that PFL3R should be most active when the fly is facing to the left of its goal—in other words, when there is a negative directional error (*θ* − *θ*_g_) (Fig. 1e), with the reverse situation holding for PFL3L.

If we neglect the contribution of PFL2 cells, then we would predict that the system’s rotational velocity commands should just resemble the right–left difference in PFL3 activity (ΣPFL3R − ΣPFL3L), which varies sinusoidally as a function of directional error (Fig. 1e). In other words, the system would behave like a classical resolver servomechanism (Fig. 1a). In this sort of mechanism, rotational velocity is nearly zero around the goal and also opposite the goal; engineers call this ‘false nulling’ because it can allow the servomechanism to settle at an angle opposite the goal (Extended Data Fig. 1). To seek this phenomenon in fly behaviour, we placed flies in a virtual-reality environment with a single prominent visual head direction cue; this environment rotated in closed loop with the fly’s rotational velocity on a spherical treadmill (Fig. 1f). The fly’s head was rigidly coupled to its body, so that heading and head direction are identical. In this type of environment, flies often follow straight paths towards a goal (Fig. 1f), with different flies adopting different goal directions (Fig. 1g); this behaviour requires an intact head direction system^25–27^. During these epochs of straight walking, we could infer the fly’s goal direction from its behavioural orientation. Every minute, we jumped the virtual environment by 90° or 180°; this often caused the fly to turn back towards its goal, implying that these jumps are perceived by the brain as head direction changes^4,25^. In agreement with our model predictions, we found that the fly’s rotational speed was generally low when it was oriented towards its goal (Fig. 1h). However, contrary to predictions, the fly’s rotational speed was high—not low—around its anti-goal, and 180° jumps evoked rotational speeds that were no lower than those evoked by 90° jumps (Extended Data Fig. 3). A model that considers only PFL3 cells cannot explain these behavioural results (Fig. 1e), suggesting an additional mechanism is recruited around the anti-goal to increase rotational speed. PFL2 cells are good candidates for this mechanism, because their population amplitude should be highest when the fly is oriented towards its anti-goal (Fig. 1e). If PFL2 cells promote high rotational speeds around the anti-goal, this would mitigate the false-nulling problem: in essence, the anti-goal is already an unstable fixed point of the system, and a mechanism that specifically increased rotational speed around the anti-goal would further destabilize that unstable fixed point, ensuring that the system could not settle there.

To summarize, we can think of these three cell populations (PFL2, PFL3R, PFL3L) as dividing the range of compass angles into three different sectors (Fig. 1i), reflecting the different shifts in their head direction inputs. Each population detects the congruence between its shifted head direction vector and a goal vector. Congruence detection is implemented by a nonlinear transformation that produces maximal output in response to maximal congruence. These outputs are then combined to generate steering commands with the appropriate direction and speed, so that small deviations from the goal are corrected with slower turns, whereas large deviations from the goal are corrected with faster turns.

## Dynamics around the anti-goal

**Extended Data Fig. 2:**
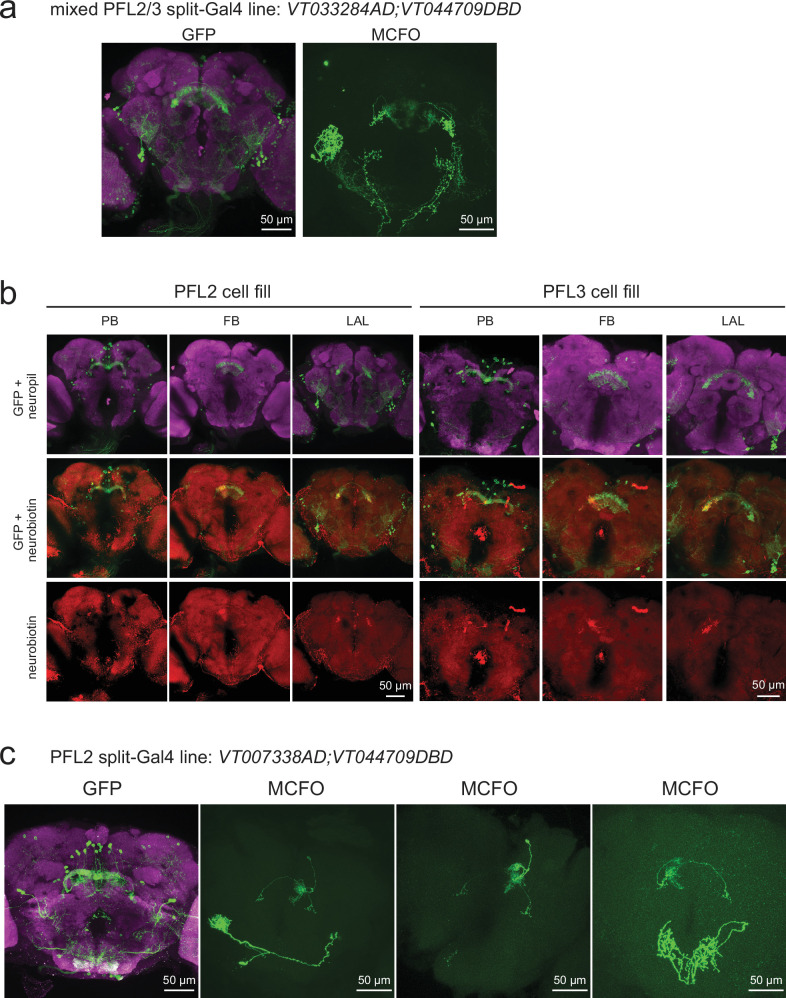
Example images for mixed and PFL2 split-Gal4 lines. **a**, Left panel shows a max z-projection of the mixed PFL2/3 split-Gal4 line expressing GFP. Right panel shows a max z-projection from an MCFO clone with 3 PFL3 neurons (PFL3 identity confirmed by counting axons) along with unidentified neurons outside of the central complex. Across 7 brains, we counted 7 PFL3 neurons and 4 PFL2 neurons, no expression from other cell types was observed in the region of the lateral accessory lobes where PFL2 and PFL3 axon terminals are found. **b**. Example cell fills obtained from electrophysiology experiments. The left 3 columns show an example PFL2 cell fill while the right 3 columns show an example PFL3 cell fill. It was common for the axonal arbors in the lateral accessory lobe (LAL) to exhibit bright fluorescence, while the dendritic arbors in the protocerebral bridge and fan-shaped body exhibited relatively dim fluorescence. The identity (PFL2, PFL3, or other) of every recorded cell was confirmed by comparing the morphology of the filled cell to the known morphology of PFL2 and PFL3 neurons; of the 30 cells recorded, 12 were verified in this manner as PFL2 cells, 15 were verified as PFL3 cells, and 3 were found to represent other cell types. **c**. Left panel shows a max z-projection of the PFL2 split-Gal4 line expressing GFP. The right three panels each show a max z-projection for three brains, each containing one PFL2 neuron. The left and rightmost of these panels also show an unidentified neuron ventral to the central complex. Across 13 brains we counted 15 PFL2 neurons, and 0 other cell types in the central complex.

To test the predictions of this model, we constructed split-Gal4 lines to target PFL2 and PFL3 cells. We were able to generate a selective PFL2 line, as well as a line targeting PFL2 and PFL3 together. We validated these lines by using genetic mosaic analysis to identify single-cell clones and then comparing these clones to morphologies from connectome data (Extended Data Fig. 2). We will focus initially on our results for PFL2 cells, as this line was the more specific line.

**Fig. 2:**
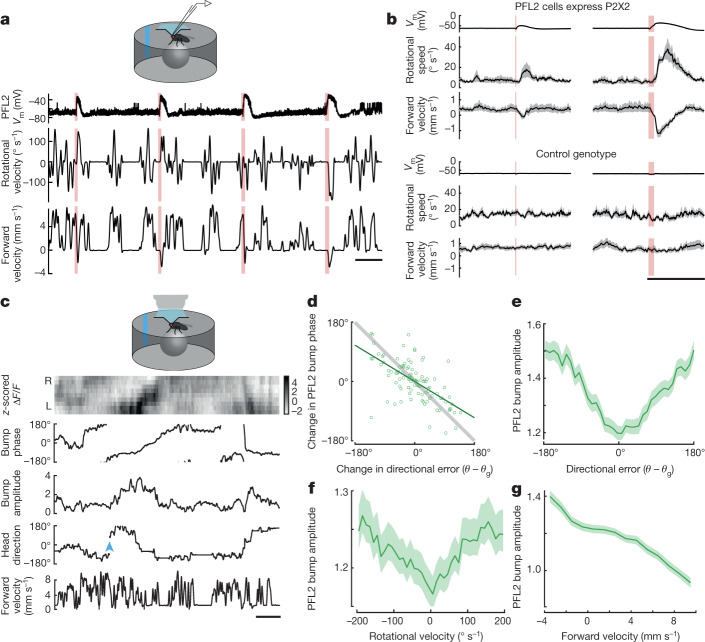
Dynamics around the anti-goal. **a**, Example experiment. ATP (red shading) depolarizes PFL2 cells expressing P2X2 (top), evoking an increase in the absolute value of rotational velocity, that is, rotational speed (middle). It also evokes a decrease in forward velocity (bottom). This fly turns right in response to the first two pulses but left in response to the last two pulses. **b**, Summary data for flies where PFL2 cells expressed P2X2 and genetic controls (mean ± s.e.m. across flies, *n* = 12 P2X2+ flies and 11 control flies). Results are shown for two ATP pulse durations (100 ms and 500 ms). See also Extended Data Fig. 4. **c**, PFL2 activity (Δ*F/F*) across the horizontal axis of the fan-shaped body over time. During this epoch, the fly is walking relatively straight. The fly’s mean head direction is taken as its goal (*θ*_g_). After the environment is jumped by 180° (blue arrowhead), the fly makes a compensatory turn to reorient towards *θ*_g_. We fit a sinusoid to Δ*F/F* at each time point to extract bump phase and amplitude. **d**, Change in PFL2 bump phase versus change in directional error. The phase of PFL2 activity moves right when the fly turns left. Each symbol denotes one time point (Pearson’s *r* = −0.63, *P* = 9 × 10^−13^), with the line of unity in grey. Shown here are data for one example fly; Extended Data Fig. 9 shows two other examples and shows the effect of *z*-scoring Δ*F/F*. **e**, PFL2 bump amplitude versus directional error (mean ± s.e.m. across flies, *n* = 33 flies). **f**, PFL2 bump amplitude versus the fly’s rotational velocity (mean ± s.e.m. across flies, *n* = 33 flies). **g**, PFL2 bump amplitude versus the fly’s forward velocity (mean ± s.e.m. across flies, *n* = 33 flies). Scale bars, 5 s (**b**), 10 s (**a**,**c**).

First, to directly activate PFL2 cells, we used a chemogenetic approach: we expressed ATP-gated ion channels (P2X2 receptors) in these cells, and we activated them specifically using iontophoresis of ATP into the protocerebral bridge, where their dendrites are located (Fig. 2a and Extended Data Fig. 4). We made a whole-cell recording from a PFL2 cell in every experiment to confirm the effects of ATP (Fig. 2a,b). At the same time, we monitored the fly’s behaviour on a spherical treadmill, again in a virtual-reality environment with a visual cue. We found that stimulating PFL2 cells generally produced turning, although the direction of the turn was often unpredictable (Fig. 2a,b). Moreover, if the fly was walking forward at the time of the stimulus, it consistently reversed direction and stepped backward (Fig. 2a,b). This response may be related to the fact that bidirectional excitation in some steering-related descending neurons is correlated with slowing or backward walking^4^. In short, PFL2 cells drive an increase in rotational movement, accompanied by a decrease in forward velocity.

Next, we used our selective PFL2 line to drive expression of GCaMP7b, and we imaged the activity of these cells with a two-photon microscope. We saw that activity in PFL2 dendrites generally formed a sinusoidal spatial pattern across the horizontal axis of the fan-shaped body (Fig. 2c). We fit a sinusoid to this pattern and extracted its phase and amplitude; we call this the ‘bump phase’ and ‘bump amplitude’. We found that the bump phase generally moved left as the fly rotated to the right (Fig. 2c,d), as expected from the anatomical inputs to PFL2 cells from the head direction system. Notably, we found that bump amplitude was minimal when the fly was oriented towards its goal and maximal around the anti-goal (Fig. 2e). Moreover, we found that high bump amplitude correlated with high rotational speed (Fig. 2f) and low forward velocity (Fig. 2g). Taken together with our chemogenetic simulation results, these data argue that PFL2 cells are recruited when the fly is facing its anti-goal, driving an increase in rotational speed, accompanied by a decrease in forward velocity. Thus, these cells provide a solution to the ‘false nulling’ problem that characterizes a classical servomechanism: they function to further destabilize the unstable fixed point in the steering control system, so that it cannot come to rest at the anti-goal.

## Dynamics around the goal

**Fig. 3:**
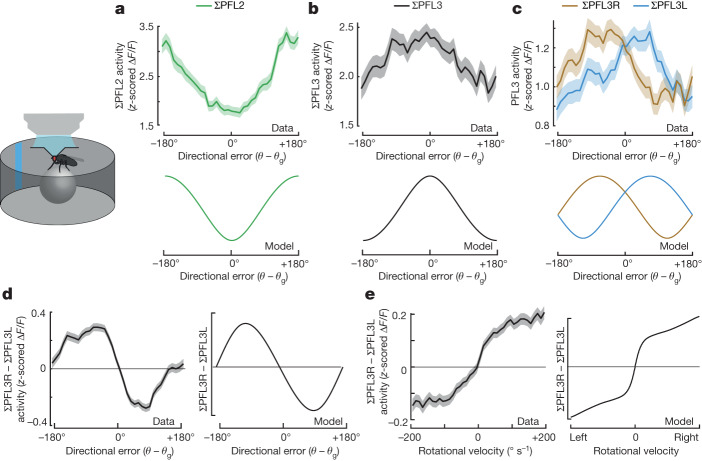
Dynamics around the goal. **a**, ΣPFL2 activity (Δ*F/F*) versus directional error (mean ± s.e.m. across flies, *n* = 33 flies). Shown here is the summed activity of the right and left PFL2 axons, where they terminate near DNa03 dendrites in the lateral accessory lobe. Model prediction is shown for comparison. **b**, ΣPFL3 activity (Δ*F/F*) versus directional error (mean ± s.e.m. across flies, *n* = 23 flies). As in **a**, the activity is summed across the right and left lateral accessory lobe, where PFL3 cells terminate onto DNa03 and DNa02. Here we used a mixed split-Gal4 line that targets PFL2 and PFL3 cells together; because our results are opposite for what we observe for PFL2 cells alone, and because our results match the predictions of our PFL3 model (shown below), we treat this as measurement of PFL3 activity (Extended Data Fig. 5). **c**, ΣPFL3R and ΣPFL3L activity in the right and left lateral accessory lobe, respectively, plotted versus directional error (mean ± s.e.m. across flies, *n* = 23 flies). Signals are imaged from our mixed split-Gal4 line but are likely dominated by PFL3, as noted above. Model predictions are shown for comparison. **d**, Right–left difference in PFL3 activity versus directional error (mean ± s.e.m. across flies, *n* = 23 flies) and model prediction. **e**, Right–left difference in PFL3 activity versus the fly’s rotational velocity (mean ± s.e.m. across flies, *n* = 23 flies) and model prediction.

Next, we imaged GCaMP7b expressed under the control of the mixed split-Gal4 line that targets both PFL2 and PFL3 cells (Extended Data Fig. 2). Here, rather than imaging the dendritic arbours, we focused on the lateral accessory lobes, where PFL2 and PFL3 axons terminate, in order to separate PFL3L from PFL3R. PFL2 and PFL3 axon terminals are intermingled in the lateral accessory lobes, but we found that calcium signals in the mixed line were quite different from the signals we observed in PFL2 cells. In the PFL2-specific line, calcium signals in the lateral accessory lobes were generally maximal around the anti-goal (Fig. 3a), as we would expect from our imaging data from their dendritic arbours (Fig. 2e). However, in the mixed line, we saw the opposite: calcium signals in the lateral accessory lobes were generally maximal around the goal (Fig. 3b); this is what the model predicts for the PFL3 populations, and it implies that the signals in the mixed line are dominated by PFL3 rather than PFL2. This could be due to stronger Gal4 expression in PFL3 versus PFL2, or other differences between these cell types. Regardless, this result implies that we can treat the right and left lateral accessory lobe signals as a readout of the summed activity of each PFL3 population (ΣPFL3R and ΣPFL3L).

In agreement with model predictions, we found that ΣPFL3R is highest when the fly is just to the left of its goal, and vice versa for ΣPFL3L (Fig. 3c).The right–left difference between these signals is a roughly sinusoidal function of the fly’s orientation relative to its goal, supporting the predictions of the model (Fig. 3d). Moreover, we found that the right–left difference was predictive of the fly’s rotational velocity, again consistent with the model (Fig. 3e) and consistent with the idea that these cells drive rotational velocity changes. This differs from what we see in our PFL2-specific line, where axonal projections are symmetrically active regardless of head direction, as we would predict based on PFL2 anatomy (Extended Data Fig. 5).

In summary, our data argue that PFL3 cells drive directional steering manoeuvres that correct small deviations from the fly’s intended path. PFL3R cells are most active when the fly is oriented just to the left of its goal, and the reverse is true for PFL3L. Finally, right–left differences in PFL3 activity are predictive of rotational velocity, consistent with the direct excitatory projections of these cells to steering-related descending neurons.

## Mechanisms underlying network dynamics

**Fig. 4:**
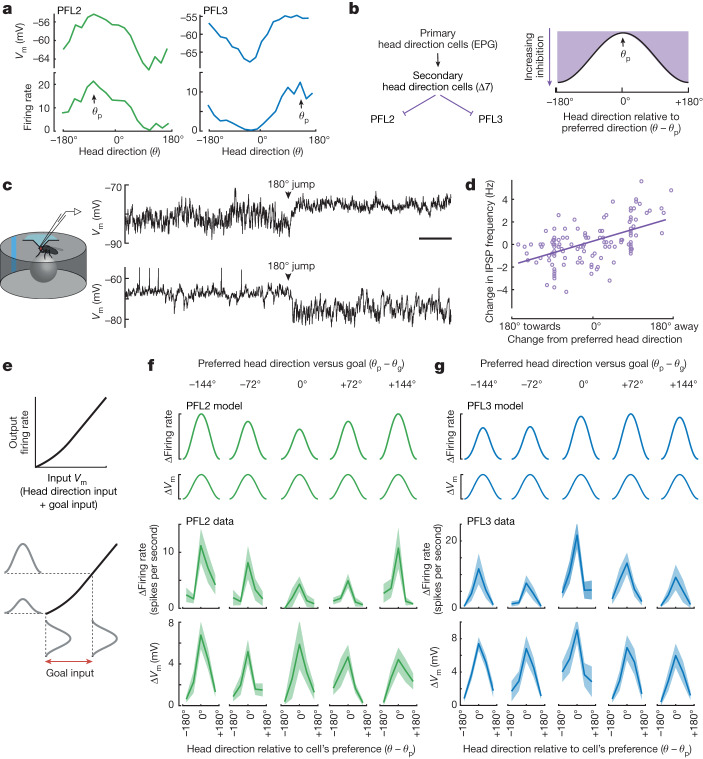
Navigation dynamics at cellular resolution. **a**, Head direction tuning in an example PFL2 cell and an example PFL3 cell. Preferred direction is *θ*_p_. **b**, Each PFL2 and PFL3 cell is predicted to receive synaptic inhibition that varies sinusoidally with head direction. **c**, Whole-cell recordings from PFL2 and PFL3 cells showing changes in IPSP frequency when we impose a rotational jump on the virtual environment, emulating a change in *θ*. **d**, Change in IPSP frequency versus change in *θ* (relative to *θ*_p_, mean ± s.e.m. across cells, *n* = 12 PFL3 and 10 PFL2 cells in 22 flies, Pearson’s *r* = 0.53). The effect of *θ* is significant (*P* = 8 × 10^−3^, two-way ANOVA, with *θ* and fly identity as the two factors). This analysis uses time points when the fly was standing still, because this makes individual IPSPs more clearly detectable. **e**, Model: a nonlinearity transforms *V*_m_ into firing rate for each model cell. Each cell receives head direction input that is cosine tuned to (*θ* − *θ*_p_). The goal cell input to each cell represents a bias that does not change with head direction. This bias moves the cell’s input along the nonlinear function, changing the amplitude of the firing rate tuning curve. **f**, PFL2 cells are divided into bins based on (*θ*_p_ − *θ*_g_). For each cell, we subtract the minimum *y*-axis value in the tuning curve, then we compute the mean of cells in the bin, for both firing rate and *V*_m_. Model output (top) is compared with data (bottom, *n* = 11 cells, mean ± s.e.m. across cells). **g**, Same but for PFL3 neurons (*n* = 15 cells, mean ± s.e.m. across cells). Here we combine results from PFL3R and PFL3L (after reversing the left–right order of the five bins for the PFL3L cells, so that the model outputs are identical for R and L). Scale bar, 2 s.

Next, to understand the inputs to PFL2 and PFL3 cells, we performed genetically targeted in vivo patch-clamp recordings. In line with model predictions, we found that individual PFL2 and PFL3 cells are often strongly tuned to head direction (Fig. 4a), with different cells having different preferred directions (*θ*_p_). Connectome data indicate that PFL2 and PFL3 cells receive some direct synaptic input from primary head direction cells (EPG cells) but that they receive most of their head direction input (about 80%) from secondary head direction cells, called Δ7 cells^5^. Because Δ7 cells are glutamatergic, and because glutamate is largely an inhibitory neurotransmitter in the *Drosophila* brain^28,29^, we would expect that the majority of the head direction input to PFL2 and PFL3 cells would arrive in the form of synaptic inhibition (Fig. 4b). Indeed, we found that PFL2 and PFL3 cells are bombarded by inhibitory postsynaptic potentials (IPSPs) whose frequency depends on head direction (*θ*). Jumping the virtual environment around the fly often evoked an abrupt change in IPSP frequency (Fig. 4c), with IPSP frequency increasing if the jump brought *θ* away from *θ*_p_ and IPSP frequency decreasing if the jump brought *θ* towards *θ*_p_ (Fig. 4d). These results support the conclusion that head direction tuning in PFL2 and PFL3 cells arises largely from Δ7 cells, which is important because Δ7 cells reformat the head direction signal as a spatial sinusoid^5,24^.

In the model, each PFL2 or PFL3 cell adds its head direction input with goal input, and the result is passed through a nonlinearity. From the perspective of a single PFL2 or PFL3 cell, goal input is simply a fixed bias. This bias pushes the cell’s total input up or down the nonlinearity, thereby changing the amplitude of the head direction tuning curve (Fig. 4e). In the model PFL2 population, the goal input that each cell receives increases as the cell’s preferred head direction *θ*_p_ moves away from the goal direction *θ*_g_ (Fig. 1d), and so cells with *θ*_p_ near the anti-goal have the largest-amplitude head direction tuning curves; indeed, our electrophysiological data confirm this prediction (Fig. 4f). Conversely, in the model PFL3 population, goal input is largest for cells whose preferred head direction *θ*_p_ is shifted just counterclockwise or clockwise from *θ*_g_ (for PFL3R or PFL3L, respectively; Fig. 1d). These should be the cells with the largest-amplitude head direction tuning curves, and again our data confirm this prediction (Fig. 4g); an independent study of PFL3 cells reached a similar conclusion^30^. Interestingly, we only find these effects at the level of spike rate; we do not see these trends at the level of the cell’s membrane potential (Fig. 4f,g and Extended Data Fig. 6); this finding implies that the nonlinearity in the model is implemented by the voltage-gated conductances that transform membrane potential to spiking.

## Modulating the scale of network activity

Our data indicate that PFL2 cells specifically boost steering gain around the anti-goal. But why would it be useful for this boost to be restricted to head directions around the anti-goal? Why not steer with high gain at all times?

**Fig. 5:**
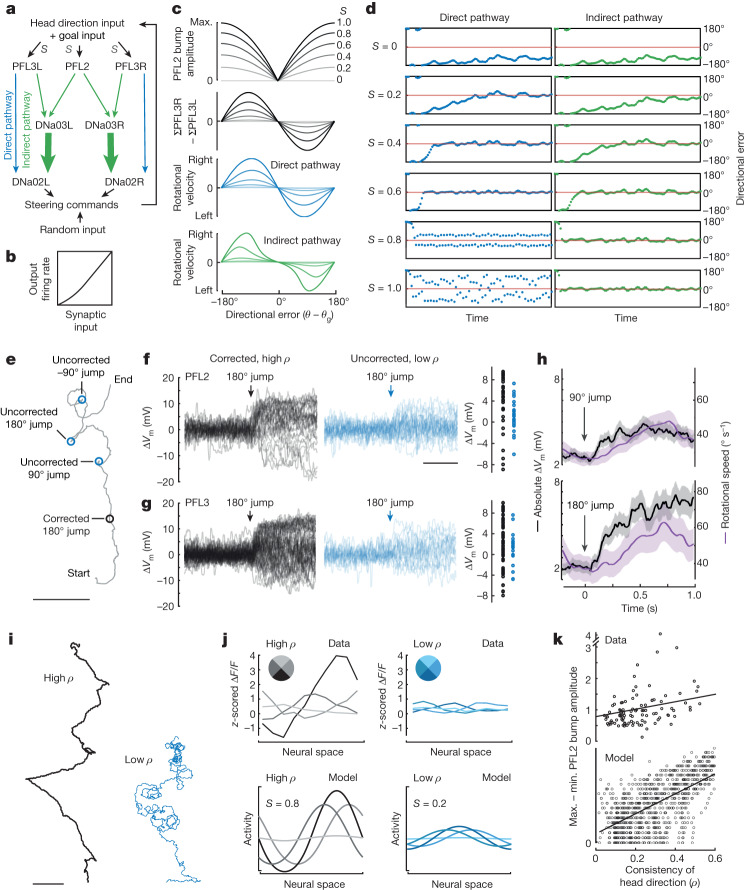
Modulating the scale of network activity. **a**, Direct and indirect pathways. *S* adjusts the magnitude of total input to PFL2 and PFL3 cells. **b**, Nonlinear activation function. **c**, Top, model PFL2 bump amplitude and (ΣPFL3R − ΣPFL3L) versus directional error. Bottom, rotational velocity produced by the direct or indirect pathway alone. With both pathways, results are similar to the indirect pathway alone. **d**, Model: directional error over time. As *S* increases, the network brings head direction towards the goal (red line). If the indirect pathway is omitted, high *S* produces overshooting. **e**, Data: example path during four jumps of the virtual environment, separated by 60 s. The fly corrects for the first jump, but not the rest. The probability of correction typically did not change over time. **f**, Change in PFL2 membrane potential (Δ*V*_m_) before and after each 180° jump, comparing corrected jumps with high *ρ* (*n* = 31 of 276 jumps) or uncorrected jumps with low *ρ* (*n* = 27 of 276 jumps). Variance in Δ*V*_m_ is higher for corrected versus uncorrected jumps (*P* = 0.01363, Brown–Forsythe test). See also Extended Data Fig. 7. **g**, Same but for PFL3 (*n* = 60 of 348 corrected, 17 of 348 uncorrected, *P* = 0.02776). **h**, Absolute Δ*V*_m_ and rotational speed during corrected jumps. Mean ± s.e.m., *n* = 157 of 701 (90°) and *n* = 91 of 624 (180°), pooling data from PFL2 and PFL3 cells. **i**, Path of two flies in a virtual environment over 10 min, one with high consistency of head direction (high *ρ*) and the other with low *ρ*. **j**, Spatial profile of PFL2 activity, divided into four bins based on head direction, relative to the directions associated with the highest and lowest PFL2 bump amplitude (darkest and lightest traces, respectively). Data (top) are from the two paths in **i**. Model results (bottom) are generated by setting *S* = 0.8 or *S* = 0.2, producing high or low *ρ*, respectively, as shown in **d**. **k**, Data (top): for each 10 min trial we computed *ρ* and also analysed the spatial profile of PFL2 activity as in **j**, taking the difference between the maximum and minimum bump amplitudes. Across trials (symbols), bump amplitude modulation is correlated with *ρ* (Pearson’s *r* = 0.37096, *P* = 1.7 × 10^−4^, *n* = 33 flies). Model (bottom): same analysis on model output. Here we obtained a range of model outcomes by varying *S* and using different random seeds. Scale bars, 5 cm (**e**,**i**), 1 s (**f**). In **k**, Max., maximum; min., minimum.

To develop an intuition for this issue, we modelled the relationship between PFL2 and PFL3 activity and steering. PFL3 cells synapse directly onto descending neurons (DNa02; Fig. 5a), and the right–left difference in DNa02 activity is linearly proportional to the fly’s subsequent rotational velocity^4^. Meanwhile, PFL3 cells also synapse onto DNa03, which is one of the strongest inputs to DNa02 in the brain^4,5,31,32^; we call this the ‘indirect pathway’ (Fig. 5a). Each DNa03 cell also receives input from every PFL2 cell. In the model, DNa03 sums PFL3 and PFL2 input and then passes this sum through a nonlinear activation function (Fig. 5b). Note that each PFL2 axon projects bilaterally, but it can still influence steering in our model, because it creates an excitatory drive that pushes DNa03 output towards the steeper part of its nonlinear activation function, amplifying the right–left asymmetry that DNa03 inherits from PFL3. DNa02 then sums PFL3 and DNa03 input (from the direct and indirect pathway, respectively), and this sum is again passed through the same nonlinearity. We add a small random component to the steering signal, to account for noise and other factors influencing steering, and we feed the resulting steering commands back into the head direction system, thereby closing the loop for feedback control.

A free parameter in this model is the scalar value (*S*) that controls the overall magnitude of the synaptic input to PFL2 and PFL3 cells (Fig. 5a), and thus the strength of the downstream steering commands evoked by any given head direction (Fig. 5c). If *S* is too low, feedback is slow to correct deviations from the goal. Conversely, if *S* is too high, the system overshoots the goal and tends to oscillate. With the direct pathway alone, *S* must be tuned within a narrow range of values to avoid these outcomes, but with the indirect pathway, there is a wider range of acceptable values (Fig. 5d) because the indirect pathway has high gain around the anti-goal but low gain around the goal (Fig. 5c). In short, the indirect pathway in general and PFL2 cells in particular function to manage the tradeoff between speed and accuracy, favouring speed for large errors, but accuracy for small errors.

This model illustrates how variations in *S* can produce variations in the vigour of goal-directed steering. In fact, in our data, we noticed variations in the vigour of goal-directed steering: we observed vigorous corrective steering after some jumps of the virtual environment, but no corrective steering after other jumps. Jumps that triggered corrective steering during epochs of high head direction consistency (high *ρ*) produced larger changes in PFL2 and PFL3 membrane potential, as compared to uncorrected jumps that occurred during epochs of low head direction consistency (low *ρ*, Fig. 5e–g). This observation suggests that the brain regulates the scale of the synaptic inputs to PFL2 and PFL3 cells as a way to modulate the strength of goal-directed steering. Importantly, jump-evoked changes in membrane potential preceded steering (Fig. 5h and Extended Data Fig. 7), supporting the idea that PFL2 and PFL3 cells are causal for steering.

We also quantified head direction consistency (*ρ*) over long time epochs (Fig. 5i). During epochs of high *ρ*, our imaging data revealed that the amplitude of the PFL2 bump depended strongly on head direction, and indeed our model predicts this as a consequence of high *S* (Fig. 5j,k). Conversely, during epochs of low *ρ*, the amplitude of the PFL2 bump depended only weakly on head direction, and again our model predicts this as a consequence of low *S* (Fig. 5j,k). These findings further support the idea that the brain can modulate the strength of goal-directed steering by scaling the inputs to PFL2 and PFL3 cells.

## Discussion

Whereas the brain’s maps of space are allocentric (referenced to objects in the world), motor commands are egocentric. This poses a coordinate transformation problem. Here we describe a network that solves this problem. This network creates two opponent copies of the allocentric head direction representation, with equal and opposite shifts (*θ* ± shift). Each copy is then separately compared with an allocentric goal representation, to measure congruence with the goal. The difference between the two opponent congruence values becomes an egocentric motor command. Elements of this scheme have been predicted in algorithmic models^7^ and network models^4,5,8–14^. Our data demonstrate that these theoretical predictions are largely correct, and we show that the two opponent copies are represented by the PFL3R and PFL3L populations; this conclusion is supported by an independent companion paper^30^.

At the same time, our results highlight the unexpected role of PFL2 cells. These cells provide a solution to a classic problem—namely, the fundamental tradeoff between speed and accuracy. High feedback gain allows a system to converge quickly towards its goal, and so it makes sense that gain should be high when error is large, that is, when there is a large discrepancy between the system’s current state and its goal. However, high gain can cause overshooting of the goal, especially when error is already small. We show that PFL2 cells effectively adjust the system’s gain, depending on the magnitude of the system’s current error. Specifically, PFL2 cells selectively increase the gain of steering commands around the anti-goal, where error is maximal. This allows gain to be lower around the goal, which should minimize overshooting. In this manner, PFL2 cells dynamically adjust feedback gain to match the needs of the system, a concept known as adaptive control^33^. Notably, the adaptive control exerted by PFL2 cells occurs only in the ‘indirect’ pathway, where PFL2 signals converge with PFL3 signals (Fig. 5a); the function of the ‘direct’ pathway is less clear, but it may help to initiate steering manoeuvres with minimal delay.

It is likely that there are multiple sites of adaptive gain control in this network. In particular, our data suggest that the inputs to PFL2 and PFL3 cells change in scale over time (which we model as changes in the parameter *S*); this may provide a way to modulate the organism’s commitment to remembered or internalized goals. For example, *S* might increase when the organism needs to be moving vigorously towards a high-value remembered goal; conversely, *S* might decrease when the organism needs to be more open to exploration of the local environment. Mechanistically, this modulation could be implemented by inhibitory tangential cell inputs to the fan-shaped body that are well-positioned to shunt the inputs to PFL2 and PFL3 dendrites, and it could explain why, in other insect species, these cells sometimes show unusually weak responses to head direction changes^22^. Alternatively, the strength of goal-directed steering could be altered by modulating the amplitude of goal cell output (Extended Data Fig. 8).

In the future, it will be interesting to investigate how and where goals are written into memory. The companion paper to this study identifies one goal cell population^30^, but there are dozens of candidate goal cell types in the fan-shaped body with the appropriate anatomy to represent a goal as a spatial sinusoid^5,9,12,13^. In principle, multiple goals could be stored as spatial patterns of persistent activity or synaptic weights.

This network also suggests a solution to the problem of representational drift^34–36^. As the phase of the head direction representation drifts over time during spatial learning^37–39^, the same process that first initialized the goal representation could continually update that representation, to keep it aligned with the coordinate frame of the head direction system. As a result, motor commands would be protected from drift, which might explain why representational drift is less obvious in cells more strongly correlated with motor performance^40^.

In summary, our results reveal how the sense of direction can be used to generate locomotor commands with adaptive gain that manages the tradeoff between speed and accuracy. Our conclusions generate testable predictions for how goals could be stored in memory, retrieved on demand, modulated by context and protected from drift. Because the basic problems of navigation are fundamental problems of geometry and information retrieval, the solutions we describe here may have general relevance for other systems.

## Methods

### Flies

Unless otherwise specified, flies were raised on cornmeal-molasses food (Archon Scientific) in an incubator on a 12 h:12 h light:dark cycle at 25 °C at 50–70% relative humidity. Experimenters were not blinded to fly genotype. For iontophoresis stimulus experiments (Fig. 2a,b) flies were grouped for analysis based on genotype. Sample sizes were chosen based on conventions in our field for standard sample sizes; these sample sizes are conventionally determined on the basis of the expected magnitude of animal-to-animal variability, given published results and pilot data. All experiments used flies with at least one wild-type copy of the *white* (*w*) gene. Genotypes used in each figure are as follows.

Fig. 1:

PFL2 and PFL3 calcium imaging, w/+;P{VT007338-p65ADZp}attP40/20XUAS-IVS-cyRFP{VK00037}; P{y[+t7.7] w[+mC]=VT044709-GAL4.DBD}attP2/PBac{y[+t7.7] w[+mC]=20XUAS-IVS-jGCaMP7b}VK00005.

PFL2 calcium imaging, w/+;P{VT033284-p65AD}attP40/20XUAS-IVS-cyRFP{VK00037}; P{y[+t7.7];P{VT007338-Gal4DBD}attP2/PBac{y[+t7.7] w[+mC]=20XUAS-IVS-jGCaMP7b}VK00005.

Fig. 2:

PFL2 cells expressing P2X2, w/+;P{VT007338-p65ADZp}attP40/P{w[+mC]=UAS-Rnor\P2rx2.L}4/;P{y[+t7.7] w[+mC]=VT044709-GAL4.DBD}attP2/20XUAS-mCD8::GFP {attP2}.

Empty split control,

w/+;P{y[+t7.7] w[+mC]=p65.AD.Uw}attP40/P{w[+mC]=UAS-Rnor\P2rx2.L}4;P{y[+t7.7] w[+mC]=GAL4.DBD.Uw}attP2/20XUAS-mCD8::GFP {attP2}.

PFL2 calcium imaging,

w/+;P{VT007338-p65ADZp}attP40/20XUAS-IVS-cyRFP{VK00037}; P{y[+t7.7] w[+mC]=VT044709-GAL4.DBD}attP2/PBac{y[+t7.7] w[+mC]=20XUAS-IVS-jGCaMP7b}VK00005.

Fig. 3:

PFL2 calcium imaging,

w/+;P{VT007338-p65ADZp}attP40/20XUAS-IVS-cyRFP{VK00037}; P{y[+t7.7];P{y[+t7.7] w[+mC]=VT044709-GAL4.DBD}attP2/PBac{y[+t7.7] w[+mC]=20XUAS-IVS-jGCaMP7b}VK00005.

PFL2 and PFL3 calcium imaging,

w/+;P{VT033284-p65AD}attP40/20XUAS-IVS-cyRFP{VK00037}; P{y[+t7.7];w[+mC]=VT044709-GAL4.DBD}attP2/PBac{y[+t7.7] w[+mC]=20XUAS-IVS-jGCaMP7b}VK00005.

Fig. 4:

w/+;P{VT033284-p65AD}attP40/P{20XUAS-IVS-mCD8::GFP}attP40;P{y[+t7.7] w[+mC]=VT044709-GAL4.DBD}attP2/+.

Fig. 5:

PFL2 calcium imaging,

w/+;P{VT007338-p65ADZp}attP40/20XUAS-IVS-cyRFP{VK00037}; P{y[+t7.7] w[+mC]=VT044709-GAL4.DBD}attP2/PBac{y[+t7.7] w[+mC]=20XUAS-IVS-jGCaMP7b}VK00005.

PFL2 and PFL3 recordings,

w/+;P{VT033284-p65AD}attP40/P{20XUAS-IVS-mCD8::GFP}attP40;P{y[+t7.7] w[+mC]=VT044709-GAL4.DBD}attP2/+.

Extended Data Fig. 2:

MCFO, w[1118] P{y[+t7.7] w[+mC]=R57C10-FLPG5}su(Hw)attP8; PBac{y[+mDint2] w[+mC]=10xUAS(FRT.stop)myr::smGdP-HA}VK00005 P{y[+t7.7] w[+mC]=10xUAS(FRT.stop)myr::smGdP-V5-THS-10xUAS(FRT.stop)myr::smGdP-FLAG}su(Hw)attP1.

PFL2 and PFL3 line:

w/+;P{VT033284-p65AD}attP40/P{20XUAS-IVS-mCD8::GFP}attP40;P{y[+t7.7] w[+mC]=VT044709-GAL4.DBD}attP2/+.

PFL2 line:

w/+;P{VT007338-p65ADZp}attP40/P{20XUAS-IVS-mCD8::GFP}attP40; P{y[+t7.7];P{y[+t7.7] w[+mC]=VT044709-GAL4.DBD}attP2)/+.

**Extended Data Fig. 3:**
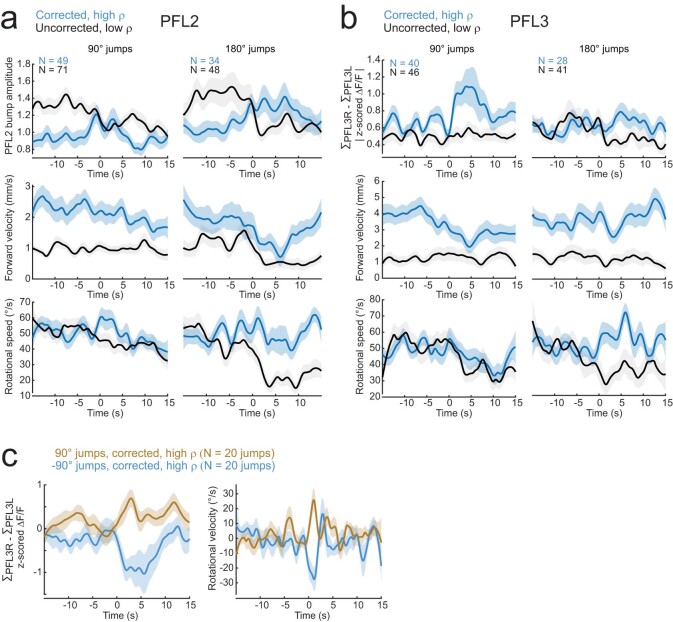
Jumps of the virtual environment during imaging experiments. **a**,. PFL2 bump amplitude, forward velocity, and rotational speed and behavior for 180° jumps and ±90° jumps of the virtual environment (mean ± s.e.m across jumps). **b**, Absolute difference in right-left PFL3 signals (|ΣPFL3R - ΣPFL3L|), forward velocity, and rotational speed and behavior for 180° jumps and ±90° jumps of the virtual environment (mean ± s.e.m across jumps). **c**, Difference in right-left PFL3 signals (ΣPFL3R - ΣPFL3L) and rotational speed, comparing +90° and −90° jumps. Clockwise (positive) jumps of the virtual environment produce higher activity in PFL3R, and rightward (positive) turns, as expected (mean ± s.e.m across jumps).

Extended Data Fig. 3:

PFL2 calcium imaging,

w/+;P{VT007338-p65ADZp}attP40/20XUAS-IVS-cyRFP{VK00037}; P{y[+t7.7] w[+mC]=VT044709-GAL4.DBD}attP2/PBac{y[+t7.7] w[+mC]=20XUAS-IVS-jGCaMP7b}VK00005.

PFL2 and PFL3 calcium imaging,

w/+;P{VT033284-p65AD}attP40/20XUAS-IVS-cyRFP{VK00037}; P{y[+t7.7];w[+mC]=VT044709-GAL4.DBD}attP2/PBac{y[+t7.7] w[+mC]=20XUAS-IVS-jGCaMP7b}VK00005.

**Extended Data Fig. 4:**
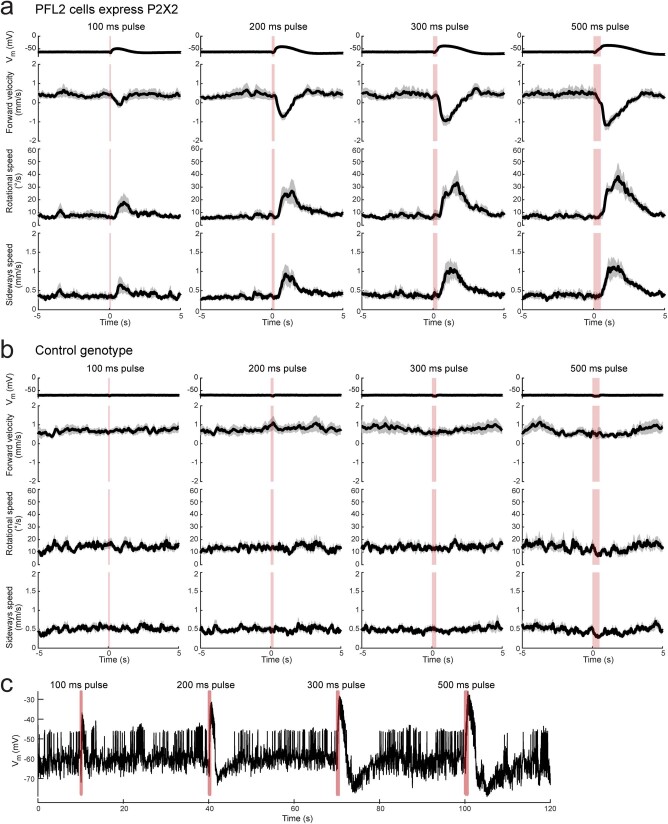
Additional data on PFL2 chemogenetic stimulation. **a**, Expanded summary data for flies where PFL2 cells expressed P2X2 showing PFL2 voltage responses to 100-ms, 200-ms, 300-ms, and 500-ms pulses of ATP, as well as simultaneously recorded locomotor activity (mean ± s.e.m across jumps). Here we show forward velocity, absolute rotational velocity (i.e., rotational speed), and absolute sideways velocity (sideways speed). **b**, Same but for genetic controls where PFL2 cells did not express P2X2 (mean ± s.e.m across jumps). **c**, Response of one example PFL2 cell to all four pulse durations over a 120-s period.

Extended Data Fig. 4:

PFL2 cells expressing P2X2, w/+;P{VT007338-p65ADZp}attP40/P{w[+mC]=UAS-Rnor\P2rx2.L}4/;P{y[+t7.7] w[+mC]=VT044709-GAL4.DBD}attP2/20XUAS-mCD8::GFP {attP2}.

Empty split control,

w/+;P{y[+t7.7] w[+mC]=p65.AD.Uw}attP40/P{w[+mC]=UAS-Rnor\P2rx2.L}4;P{y[+t7.7] w[+mC]=GAL4.DBD.Uw}attP2/20XUAS-mCD8::GFP {attP2}.

**Extended Data Fig. 5:**
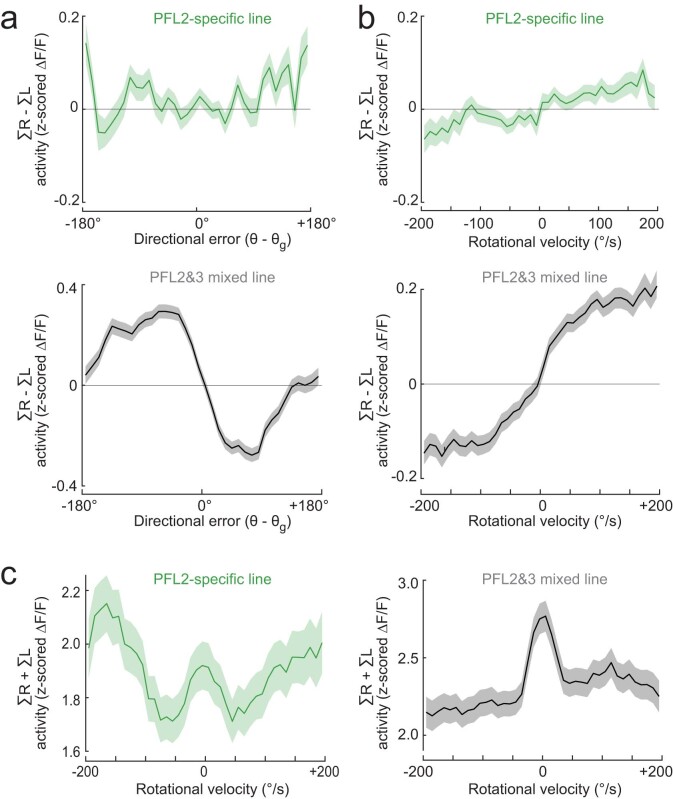
Comparing the PFL2-specific line with the PFL2&3 mixed line. **a**. Right minus left activity in the lateral accessory lobe (LAL) versus directional error, for both the PFL2-specific line and the PFL2&3 mixed line (mean ± s.e.m. n = 33 flies and n = 23 flies). **b**. Right minus left activity in the LAL versus rotational velocity, for both the PFL2-specific line and the PFL2&3 mixed line (mean ± s.e.m. n = 33 flies and n = 23 flies). **c**. Left panel: summed right and left activity in the LAL versus rotational velocity, for both the PFL2-specific line and the PFL2&3 mixed line (mean ± s.e.m. n = 33 and n = 23 flies). The dissimilarity in results for these two lines is further evidence that PFL2 cells make a minimal contribution to the mixed line. Therefore, we treat the results from the mixed line as a readout of PFL3 activity.

Extended Data Fig. 5:

PFL2 calcium imaging,

w/+;P{VT007338-p65ADZp}attP40/20XUAS-IVS-cyRFP{VK00037}; P{y[+t7.7] w[+mC]=VT044709-GAL4.DBD}attP2/PBac{y[+t7.7] w[+mC]=20XUAS-IVS-jGCaMP7b}VK00005.

PFL2 and PFL3 calcium imaging,

w/+;P{VT033284-p65AD}attP40/20XUAS-IVS-cyRFP{VK00037}; P{y[+t7.7];w[+mC]=VT044709-GAL4.DBD}attP2/PBac{y[+t7.7] w[+mC]=20XUAS-IVS-jGCaMP7b}VK00005.

**Extended Data Fig. 6:**
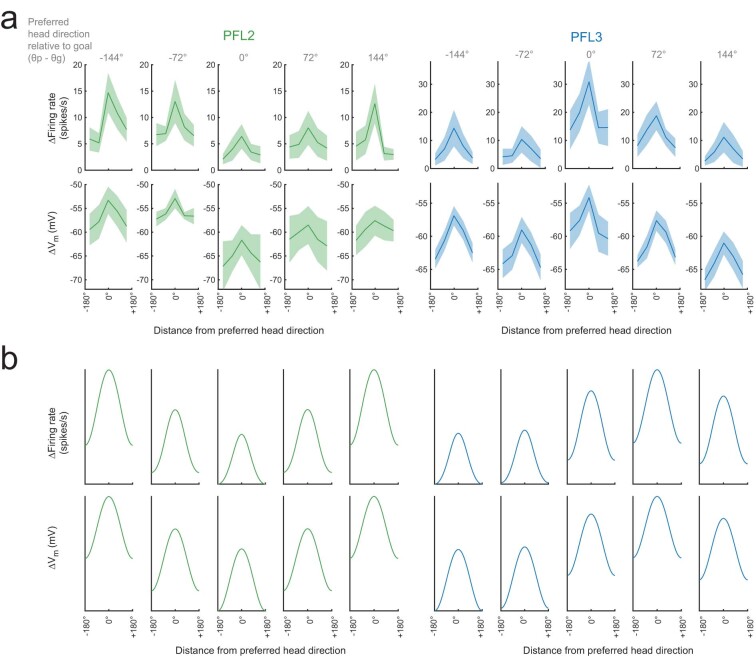
Changes in single cell head direction tuning curves depending on the distance between the cell’s preferred head direction and the goal head direction. **a**, Tuning curves for PFL2 and PFL3 cells across different distances between the cell’s preferred head direction and the goal head direction (mean ± s.e.m across jumps). The vertical offset of the cell’s head direction tuning curve changes depending on this distance but the amplitude of the tuning curves only change at the level of firing rate. **b**, The same as (a) but for model generated output. These are the same results as in Fig. 4f, g; the only difference is that we have not subtracted the minimum value of each tuning curve.

Extended Data Fig. 6:

w/+;P{VT033284-p65AD}attP40/P{20XUAS-IVS-mCD8::GFP}attP40;P{y[+t7.7] w[+mC]=VT044709-GAL4.DBD}attP2/+.

**Extended Data Fig. 7:**
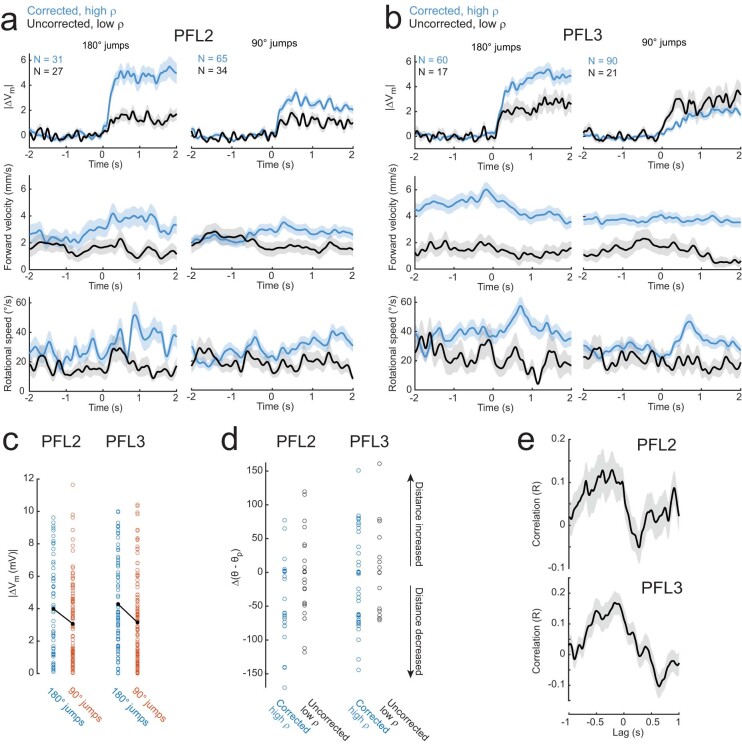
Jumps of the virtual environment during electrophysiology experiments. **a**, PFL2 recordings: absolute change in membrane potential, forward velocity, and rotational speed, for 180° jumps and ±90° jumps of the virtual environment (mean ± s.e.m across jumps, *N* is the number of jumps). **b**, Same but for PFL3. **c**, As expected, 180° jumps produce significantly larger changes in membrane potential, as compared to 90° jumps (PFL2 p = 0.041, PFL3 p = 0.0064, 2-sample, 2-tailed t-tests). **d**, Change in the difference between the fly’s head direction ($\theta$) and the cell’s preferred direction ($\theta$_p_) resulting from each cue jump in the corrected and uncorrected categories. There was no significant difference in the variance of these values between the two categories (PFL2 p = 0.68793, PFL3 p = 0.99764, Brown-Forsythe test). There was also no difference between the corrected and uncorrected categories in the mean absolute ∆($\theta$-$\theta$_p_) (PFL2 p = 0.59721, PFL3 p = 0.99723, 2-sample, 2-tailed t-tests). Thus, we might expect the two types of jumps to produce similar changes in membrane potential. The fact that we see a larger membrane potential response following a corrected jump suggests that the state of the network is different before a corrected jump, and this contributes to the behavioral response. **e**, Correlation between membrane potential and rotational speed, as a function of lag time (PFL2: n = 54, PFL3 n = 85, mean ± s.e.m across jumps). The maximum correlation is seen when we compare membrane potential with rotational speed 150−200 ms later. This is what we would expect if PFL2&3 cells are exerting a causal influence on behavior.

Extended Data Fig. 7:

w/+;P{VT033284-p65AD}attP40/P{20XUAS-IVS-mCD8::GFP}attP40;P{y[+t7.7] w[+mC]=VT044709-GAL4.DBD}attP2/+.

**Extended Data Fig. 8:**
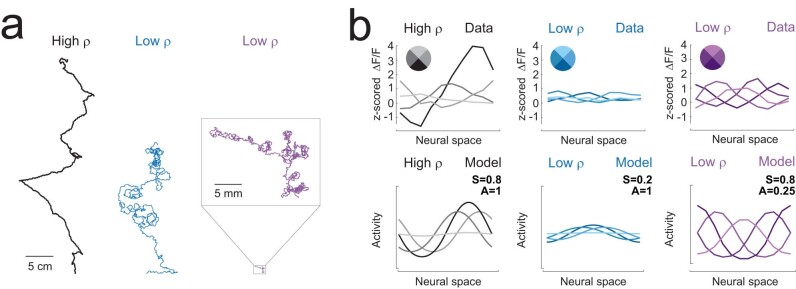
Modeling changes in behavioral state and PFL2 dynamics. **a**, Path of three flies in a virtual environment over 10 min, one with high consistency of head direction (high *ρ*) and two with low *ρ*. The third path is shown in an inset with high magnification. The first two paths are reproduced from Fig. 5i. **b**, Same as Fig. 5j, but now including data and model for the third (purple) path. Here, to model, the third path, we adjust *A* rather than *S*. Whereas *S* scales the total synaptic input to PFL2 and PFL3 cells, *A* specifically scales the amplitude of the goal signal: PFL2 = $f$(*S*·(cos($\theta$-$\theta$_0_-**h** + 180°) + *A*·cos($\theta$_g_-$\theta$_0_-**h**))) PFL3R = $f$(*S*·(cos($\theta$-$\theta$_0_-**h** + 67.5°) + *A*·cos($\theta$_g_-$\theta$_0_-**h**))) PFL3L = $f$(*S*·(cos($\theta$-$\theta$_0_-**h**-67.5°) + *A*·cos($\theta$_g_-$\theta$_0_-**h**))) where $f$ is a nonlinear function, $\theta$ is head direction, $\theta$_0_ is the angular position of the EPG bump at a head direction of 0°, $\theta$_g_ is the goal angle, and **h** is a vector with entries that tile the full 360° of angular space (equation (9), Methods). Reductions in *S* decrease the overall scale of PFL2&3 activity, without changing the dependence of bump amplitude on head direction. By contrast, reductions in *A* cause bump amplitude to become more invariant to head direction. The examples shown here suggest that changes in behavioral state may arise in some cases from changes in *S*, and in other cases from changes in *A*.

**Extended Data Fig. 9:**
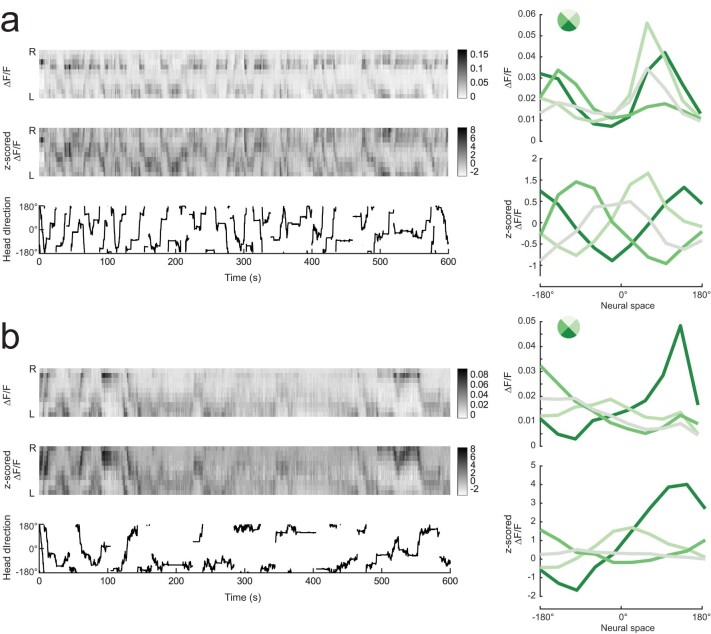
∆F/F compared to z-scored ∆F/F. **a**, Left: ∆F/F binned into 1-s increments across an entire 10-min trial for a fly that exhibited low goal fixation, before and after z-scoring, compared with head direction. Right: ∆F/F is divided into 4 bins based on the fly’s head direction relative to the head direction associated with the lowest PFL2 bump amplitude. This is done separately for raw ∆F/F (top) and z-scored ∆F/F (bottom). Here, z-scoring reduces but does not eliminate the difference in bump amplitude across bins. **b**, The same as (a) but for a fly that exhibited high goal fixation during the 10-min trial. Here, there is a strong variation in bump amplitude that persists across bins, even after z-scoring. These data are shown in Fig. 5j.

**Extended Data Fig. 10:**
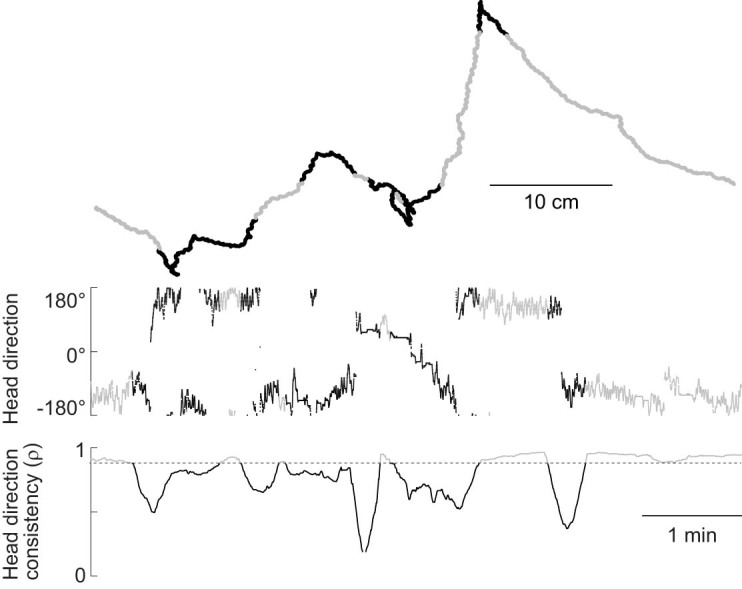
Path segmentation. To obtain an accurate estimate of the fly’s current goal direction (*θ*_g_), and thus an accurate estimate of directional error (*θ* - *θ*_g_), we needed to identify moments when *θ*_g_ might switch. We reasoned that a switch in *θ*_g_, would coincide with a dip in head direction consistency. Therefore, we looked for moments when *p* crossed a threshold value, and we broke the path into segments at those moments of threshold-crossing. This allowed us to segment a path into straight segments and to identify points where goal direction seemed to have switched. We used a threshold value of *p* = 0.88 because this produced results that corresponded to our visual impression of when the fly’s goal direction seemed to have changed, but we also confirmed that our conclusions are similar for a range of threshold values. Here we show an example path recorded over 10 min. Time points belonging to different segments are alternately shaded gray and black. Dotted line shows the threshold used to define these segments.

Extended Data Fig. 8–10:

PFL2 calcium imaging,

w/+;P{VT007338-p65ADZp}attP40/20XUAS-IVS-cyRFP{VK00037}; P{y[+t7.7] w[+mC]=VT044709-GAL4.DBD}attP2/PBac{y[+t7.7] w[+mC]=20XUAS-IVS-jGCaMP7b}VK00005.

### Origins of transgenic stocks

The following stocks were obtained from the Bloomington Drosophila Stock Center (BDSC) and previously published as follows: P{y[+t7.7]w[+mC]=VT044709-GAL4.DBD}attP2 (BDSC_75555)^41^, P{y[+t7.7] w[+mC]=p65.AD.Uw}attP40; P{y[+t7.7] w[+mC]=GAL4.DBD.Uw}attP2 (BDSC_79603), P{w[+mC]=UAS-Rnor\P2rx2.L}4/CyO (BDSC_91223)^42^, w[1118] P{y[+t7.7] w[+mC]=R57C10-FLPG5}su(Hw)attP8; PBac{y[+mDint2] w[+mC]=10xUAS(FRT.stop)myr::smGdP-HA}VK00005 P{y[+t7.7] w[+mC]=10xUAS(FRT.stop)myr::smGdP-V5-THS-10xUAS(FRT.stop)myr::smGdP-FLAG}su(Hw)attP1 (BDSC_64088)^43^.

The following stocks were obtained from WellGenetics: w[1118];P{VT007338-p65ADZp}attP40/CyO;+ (SWG9178/A), w[1118];P{VT033284-p65AD}attP40/CyO;+ (A/SWG8077). Using these lines, we constructed a split-Gal4 line whose expression in the lateral accessory lobe (LAL) is specific to PFL2 and PFL3 cells (+;P{VT033284-p65AD}attP40;P{y[+t7.7] w[+mC]=VT044709-GAL4.DBD}attP2). We validated the expression of this line using immunohistochemical anti-GFP staining and also using Multi-Color-Flip-Out (MCFO)^43^ to visualize single-cell morphologies. This line has significant non-specific expression throughout the brain but is specific for PFL2 and PFL3 in the LAL. We also constructed a split-Gal4 line to target PFL2 neurons, +;P{VT007338-p65ADZp}attP40;P{y[+t7.7] w[+mC]=VT044709-GAL4.DBD}attP2. We validated the expression of this line using immunohistochemical anti-GFP staining and also using MCFO to visualize single-cell morphologies. This line exhibits expression in various peripheral neurons but is selective for PFL2 neurons within the central complex—specifically, the protocerebral bridge, fan-shaped body and LAL.

### Fly preparation and dissection

Flies used for all experiments were isolated the day before the experiment by single-housing on molasses food. For calcium imaging experiments we used female flies 20–72 h posteclosion. For electrophysiology experiments, including the iontophoresis experiments, we used female flies 16–30 h posteclosion. No circadian restriction was imposed for the time of experiments.

Manual dissections in preparation for experiments were as follows. Flies were briefly cold-anaesthetized and inserted using fine forceps (Fine Science Tools) into a custom platform machined from black Delrin (Autotiv or Protolabs). The platform was shaped like an inverted pyramid to minimize occlusion of the fly’s eyes. The head was pitched slightly forward, so the posterior surface was more accessible to the microscope objective. The wings were removed, then the fly head and thorax were secured to the holder using UV-curable glue (Loctite AA 3972) with a brief pulse of ultraviolet light (LED-200, Electro-Lite Co.). To prevent large brain movements, the proboscis was glued in place using a small amount of the same UV-curable glue. Using fine forceps in extracellular *Drosophila* saline, a window was opened in the head cuticle, and tracheoles and fat were removed to expose the brain. To further reduce brain movement, muscle 16 was stretched by gently tugging the oesophagus, or else it was removed by clipping the muscle anteriorly. For electrophysiology and iontophoresis experiments only, the perineural sheath was minimally removed with fine forceps over the brain region of interest. For all experiments, saline was continuously superfused over the brain. *Drosophila* extracellular saline composition was: 103 mM NaCl, 3 mM KCl, 5 mM TES, 8 mM trehalose, 10 mM glucose, 26 mM NaHCO3, 1 mM NaH2PO4, 1.5 mM CaCl2 and 4 mM MgCl2 (osmolarity 270–275 mOsm). Saline was oxygenated by bubbling with carbogen (95% O_2_, 5% CO_2_) and reached a final pH of about 7.3.

### Two-photon calcium imaging

We used a two-photon microscope equipped with a galvo-galvo-resonant scanhead (Thorlabs Bergamo II GGR) and ×25, 1.10 numerical aperture (NA) objective (Nikon CFI APO LWD; Thorlabs, WDN25X-APO-MP). For volumetric imaging, we used a fast piezoelectric objective scanner (Thorlabs PFM450E). To excite GCaMP we used a wavelength-tunable femtosecond laser with dispersion compensation (Mai Tai DeepSee, Spectra Physics) set to 920 nm. GCaMP fluorescence signals were collected using GaAsP PMTs (PMT2100, Thorlabs) through a 405–488 nm band-pass filter (Thorlabs). All image acquisition and microscope control was conducted in MATLAB 2021a (MathWorks Inc), using ScanImage 2021 Premium with vDAQ hardware (Vidrio Technologies LLC) and custom MATLAB scripts for further experimental control. The region for imaging the fan-shaped body and protocerebral bridge was 150 × 250 pixels, whereas the region for imaging the LAL was 150 × 400 pixels. We acquired 10–12 slices in the *z* axis for each volume (4 µm per slice), resulting in 6–8 Hz volumetric scanning rate. For experiments using the selective PFL2 split-Gal4 line, we imaged in the protocerebral bridge, fan-shaped body, or LAL for different trials. For experiments imaging the mixed PFL2 and PFL3 split-Gal4 line, we only imaged in the LAL.

### Patch-clamp recordings

Patch pipettes were pulled from filamented borosilicate capillary glass (outer diameter: 1.5 mm, inner diameter 0.86 mm; BF150-86-7.5HP, Sutter Instrument Company), using a horizontal pipette puller (P-97, Sutter Instrument Company) to a resistance range of 9–13 MΩ. Pipettes were filled with an internal solution^44^ consisting of 140 mM KOH, 140 mM aspartic acid, 1 mM KCl, 10 mM HEPES, 1 mM EGTA, 4 mM MgATP, 0.5 mM Na_3_GTP and 15 mM neurobiotin citrate, filtered twice through a 0.22 µm PVDF filter (Millipore).

All electrophysiology experiments used a semicustom upright microscope consisting of a motorized base (Thorlabs Cerna), with conventional collection and epifluorescence attachment (Olympus BX51), but no substage optics in order to better fit the virtual-reality system. The microscope was equipped with a ×40 water immersion objective (LUMPlanFLN 40×W, Olympus) and CCD Monochrome Camera (Retiga ELECTRO; 01-ELECTRO-M-14-C Teledyne). For GFP excitation and detection, we used a 100 W Hg arc lamp (Olympus U-LH100HG) and an eGFP long-pass filter cube (Olympus F-EGFP LP). The fly was illuminated from below using a fibre optic coupled LED (M740F2, Thorlabs) coupled to a ferrule-terminated patch cable (200 µM core, 0.22 NA, Thorlabs) attached to a fibre optic cannula (200 µM core, 0.22, Thorlabs). The cannula was glued to the ventral side of the holder and positioned approximately 135° from the front of the fly to be unobtrusive to the fly’s visual field. Throughout the experiment, saline bubbled with 95% O_2_ and 5% CO_2_ was superfused over the fly using a gravity fed pump at a rate of 2 ml min^−1^. Whole-cell current-clamp recordings were performed using an Axopatch 200B amplifier with a CV-203BU headstage (Molecular Devices). Data from the amplifier were low-pass filtered using a 4-pole Bessel low-pass filter with a 5 kHz corner frequency, then acquired on a data acquisition card at 20 kHz (NiDAQ PCIe-6363, National Instruments). The liquid junction potential was corrected by subtracting 13 mV from recorded voltages^45^. Membrane potential data was then resampled to a rate of 1 kHz for ease of use and compatibility with behavioural data. To estimate baseline membrane voltage (Fig. 5e–g), we removed spikes from voltage traces by median filtering using a 50 ms window and lightly smoothed using the smoothdata function in MATLAB (loess method, 20 ms window). For all electrophysiology experiments in the mixed PFL2 and PFL3 line, we recorded from only one cell per fly. During recordings the cell was filled using internal solution containing neurobiotin citrate, so that we could visualize the cell morphology in order to determine its identity, using the protocol described in the ‘Immunohistochemistry’ section.

### Spherical treadmill and locomotion measurement

Experiments used an air-cushioned spherical treadmill and machine-vision system to track the intended movement of the animal. The treadmill consisted of a 9-mm-diameter ball machined from foam (FR-4615, General Plastics), sitting in a custom-designed concave hemispherical holder three-dimensionally printed from clear acrylic (Autotiv). The ball was floated with medical-grade breathing air (Med-Tech) through a tapered hole at the base of the holder using a flow meter (Cole Parmer). For machine-vision tracking, the ball was painted with a high-contrast black pattern using a black acrylic pen and illuminated with an IR LED (880 nm for two-photon experiments; M880L3, Thorlabs, or 780 nm for electrophysiology experiments; M780L3, Thorlabs). Ball movement was captured online at 60 Hz using a CMOS camera (CM3-U3-13Y3M-CS for two-photon imaging, or CM3-U3-13Y3C-CS for electrophysiology, Teledyne FLIR) fitted with a macro zoom lens InfiniStix (68 mm ×0.66 for two-photon, InfiniStix 94 mm ×0.5 for electrophysiology). The camera faced the ball from behind the fly (at 180°). Machine vision software (FicTrac v.2.1) was used to track the position of the ball^43^ in real time. We used a custom Python script to output the forward axis ball displacement, yaw axis ball displacement, forward ball displacement and gain-modified yaw ball displacement to an analogue output device (Phidget Analog 4-Output 1002_0B) and recorded these signals along with other experimental timeseries data on a data acquisition card (NiDAQ PCIe-6363) card at 20 kHz. The gain-modified yaw ball displacement voltage signal was also used to update the azimuthal position of the visual cues displayed by the visual panorama.

### Visual panorama and visual stimuli

To display visual stimuli, we used a circular panorama built from modular square (8 × 8 pixel) LED panels^46^. The circular arena was twelve panels in circumference and two panels tall. To accommodate the ball-tracking camera view and the light source the upper panel 180° behind the fly was removed. In all experiments, the modular panels contained blue LEDs with peak blue (470 nm) emission; blue LEDs were chosen to reduce overlap with the GCaMP emission spectrum. For calcium imaging experiments, four layers of gel filters were added in front of the LED arena (Rosco, R381) to further reduce overlap in spectra. For electrophysiology experiments, only two layers of gel filters were used. On top of the gel filters in both cases we added a final diffuser layer to prevent reflections (SXF-0600, Snow White Light Diffuser, Decorative Films). The visual cue was a bright (positive contrast) 2-pixel-wide (7.5°) vertical bar. The bar’s height was the full two-panel height of the area (except for −165 to +165° behind the fly with a single visual display panel, where the bar was half this height). The bar intensity was set at a luminance value of 4 with a background luminance of 0 (maximum value 15).

The azimuthal position of the cue was controlled during closed-loop experiments by the yaw motion of the ball (see section ‘Spherical treadmill and locomotion measurement’). For all experiments, a yaw gain of 0.7 was used, meaning that the visual cue displacement was 0.7 times the ball’s yaw displacement. For calcium imaging and electrophysiology experiments the cue was instantaneously jumped every 60 s by ±90° or 180°. Immediately following each jump, the cue would continue to move in closed loop with the fly’s movements. We recorded the position of the cue during experiments using analogue output signals from the visual panels along with other experimental timeseries data on a data acquisition card at 20 kHz (PCIe-6363, National Instruments). We converted analogue signals from the visual panels into cue position in pixels during offline analysis. Cue positions were then converted into head direction as follows: 0° when the fly was directly facing the cue, 90° when the fly’s head direction was 90° clockwise to the cue, −90° when the fly was 90° counterclockwise and 180° when the fly was facing directly away from the cue. These signals were lightly smoothed and values above 180° or below −180° were set to ±180°.

### Experimental trial structure

Before data collection in each experiment, the fly walked for a minimum of 15 min in closed loop with the visual cue. For calcium imaging experiments, data were collected in 10 min trials. In each trial, the fly was in closed loop with the cue, and every 60 s the cue jumped to a new location relative to its current one, alternating between +90°, 180° and −90°, in that order. Between trials during calcium imaging experiments, there was 30 s of darkness. Electrophysiology experiments followed a similar protocol, though occasionally 20 min trials were collected rather than 10 min trials. Additionally, during the intertrial period, flies viewed the cue in closed loop. As these experiments were heavily dependent on spontaneously performed behaviour, trials were run until the fly stopped walking or, in the case of electrophysiology experiments, the cell recording quality significantly decreased.

### Iontophoresis stimuli

Pipettes for iontophoresis were pulled from aluminosilicate capillary glass (outer diameter 1.5 mm, inner diameter 1.0 mm, Sutter Instrument Company) to a resistance of approximately 75 MΩ using a horizontal pipette puller (P-97, Sutter Instrument Company). Pipettes were filled with a solution^47^ consisting of 10 mM ATP disodium in extracellular saline with 1 mM AlexFluor 555 hydrazine (Thermo Fisher Scientific) for visualization. This solution was stored in aliquots at −20 °C, thawed fresh daily and kept on ice during the experiment. The tip of the iontophoresis pipette was positioned to be approximately in the medial region of the protocerebral bridge every trial. During experimental trials, we simultaneously recorded from a PFL2 neuron. During control trials, we recorded from unidentified neurons with somata in the same approximate region as PFL2 somata (medial area dorsal to the protocerebral bridge). Pulses of ATP were delivered using a dual current generator iontophoresis system (Model 260, World Precision Instruments). Holding current was set to 10 nA to prevent solution leakage, and a current of −200 nA was used for ejection. Visual confirmation of ATP ejection following current pulses was obtained before and after each trial. For the duration of the 10 min trial period, flies viewed a visual cue that moved in closed loop with their rotational movements, as described above. Throughout the trial, pulses were delivered every 30 s with lengths of 100, 200, 300 and 500 ms, repeating in that order.

### Immunohistochemistry

Brains were dissected from female flies 1–3 days posteclosion in *Drosophila* external saline and fixed in 4% paraformaldehyde (Electron Microscopy Sciences, catalogue no. 15714) in phosphate-buffered saline (PBS, Thermo Fisher Scientific, 46-013-CM) for 15 min at room temperature. Brains were washed with PBS before adding a blocking solution containing 5% normal goat serum (Sigma-Aldrich, catalogue no. G9023) in PBS with 0.44% Triton-X (Sigma-Aldrich, catalogue no. T8787) for 20 min. Brains were then incubated in primary antibody with blocking solution for roughly 24 h at room temperature, washed in PBS and incubated in secondary antibody with blocking solution for roughly 24 h at room temperature. Primary and secondary antibodies were protocol-specific (see below). Brains were then rinsed with PBS and mounted in antifade mounting medium (Vectashield, Vector Laboratories, catalogue no. H-1000) for imaging. For MCFO protocols, a tertiary incubation step for about 24 h at room temperature and wash with PBS was performed before mounting. Mounted brains were imaged on a Leica SPE confocal microscope using a ×40, 1.15 NA oil-immersion objective. Image stacks comprised 50 to 200 *z*-slices at a depth of 1 μm per slice. Image resolution was 1,024 × 1,024 pixels. For visualizing Gal4 expression patterns, the primary antibody solution contained chicken anti-GFP (1:1,000, Abcam, catalogue no. ab13970) and mouse anti-Bruchpilot (1:30, Developmental Studies Hybridoma Bank, nc82). The secondary antibody solution contained Alexa Fluor 488 goat anti-chicken (1:250, Invitrogen, catalogue no. A11039) and Alexa Fluor 633 goat anti-mouse (1:250, Invitrogen, catalogue no. A21050). For visualizing cell fills after whole-cell patch-clamp recordings, 1:1,000 streptavidin::Alexa Fluor 568 (Invitrogen, catalogue no. S11226) was added to the primary and secondary solutions.

For MCFO^48^, the primary antibody solution contained mouse anti-Bruchpilot (1:30, Developmental Studies Hybridoma Bank, nc82), rat anti-Flag (1:200, Novus Biologicals, catalogue no. NBP1-06712B) and rabbit anti-HA (1:300, Cell Signaling Technology, catalogue no. 3724S). The secondary antibody solution contained Alexa Fluor 488 goat anti-rabbit (1:250, Invitrogen, catalogue no. A11039), ATTO 647 goat anti-rat (1:400, Rockland, catalogue no. 612-156-120) and Alexa Fluor 405 goat anti-mouse (1:500, Invitrogen, catalogue no. A31553). The tertiary antibody solution contained DyLight 550 mouse anti-V5 (1:500, Bio-Rad, catalogue no. MCA1360D550GA).

### Processing calcium imaging data

Analysis was performed in either MATLAB 2019 or MATLAB R2021a. The calcium imaging dataset comprised 23 flies expressing GCaMP under the control of the PFL3 + 2 split-Gal4 line and 33 flies expressing GCaMP under the control of the PFL2 split-Gal4 line. Rigid motion correction in the *x*, *y* and *z* axes was performed for each trial using the NoRMCorre algorithm^49^. Each region of interest (ROI) was defined across the *z*-stack. For each ROI Δ*F/F* was calculated with the baseline fluorescence (*F*) defined as the mean of the bottom 10% of fluorescence values in a given trial (600 s in length). From this measurement a modified *z*-score was calculated using the median absolute deviation (MAD) normalized difference from the median, which we refer to as the *z*-scored Δ*F/F* (Extended Data Fig. 9):

1 $${y}_{i}=\frac{{x}_{i}-\,\underline{X}}{{\rm{MAD}}},{\rm{where}}\,\underline{X}={\rm{median}}\,{\rm{of}}\,X,\,{\rm{MAD}}={\rm{median}}\,(| {x}_{i}-\underline{X}| )$$

For protocerebral bridge imaging, ten ROIs were defined, one for each of the ten glomeruli occupied by PFL2 dendrites and defined to be approximately the same width and without overlap, constrained by estimated anatomical boundaries. For fan-shaped-body imaging, nine ROIs were defined for PFL2 neurites corresponding to the nine columns spanning the horizontal axis of the fan-shaped body. ROIs were approximately the same width without overlap. For LAL imaging, two ROIs were defined, one for the left LAL and one for the right. In any given 10 min epoch, we imaged either the protocerebral bridge or the fan-shaped body, or the LAL, that is, one brain region only. Signals in the protocerebral bridge and fan-shaped body had a similar sinusoidal profile, similar bump amplitude and a similar relationship to fly behaviour, so we used both protocerebral-bridge-imaging epochs and fan-shaped-body-imaging epochs to obtain our measurements of bump amplitude, and we pooled these bump amplitude measurements without regard to whether they came from the protocerebral bridge or fan-shaped body, see Figs. 2e–g, 3a and 5k, and Extended Data Figs. 3 and 5. The single fly examples shown in Fig. 5j, and Extended Data Figs. 8 and 9 are from trials where we imaged the protocerebral bridge.

### Processing locomotion and visual arena data

The position of the spherical treadmill was computed online using machine vision software (Fictrac v.2.1) and output as a voltage signal for acquisition. For post hoc analysis, the voltage signal was converted into radians and unwrapped. Signals were then low-pass filtered using a second-order Butterworth filter with 0.003 corner frequency and downsampled to half the ball-tracking update rate.Velocity was calculated using the MATLAB gradient function. Artefactually large velocity values (greater than 20 rad s^−1^) were set to 20 rad s^−1^, and timeseries were then smoothed using the smooth function in MATLAB (using the loess method with an 33 ms window) and resampled to 60 Hz, the ball-tracking update rate. Forward and sideways velocities were then converted to millimetres per second while yaw (rotational) velocity was converted to degrees per second.

During calcium imaging, we acquired a signal from our imaging software indicating the end of each volumetric stack on the same acquisition card as online ball tracking signals. These imaging time points were then resampled to the ball-kinematic data update rate of 60 Hz, allowing us to align the acquired volumes. Electrophysiology data were collected on the same acquisition card as online ball tracking signals, so alignment was not required; however, ball-tracking data were resampled to 1 kHz to match the sampling rate of the electrophysiology data.

### Computing inferred goal direction and consistency of head direction across trials

Head direction (*θ*) and consistency of head direction (*ρ*) were calculated for every datapoint over each entire trial using a 30 s window centred on each datapoint index. Here we excluded datapoints where the fly’s cumulative speed (forward + sideways + rotational) was less than 0.67 rad s^−1^. At values below this threshold, the fly is essentially standing still, so including these time points might result in an overestimation of the fly’s internal drive to maintain its head direction. We also excluded time points within 5 s after a cue jump; this was to avoid underestimating the fly’s internal drive to maintain its head direction, as these points represent a forced deviation from the angle the flies were attempting to maintain. If no datapoints within the 30 s window satisfied these requirements, then the window was excluded from further analyses. Head directions were treated as unit vectors and used to compute the goal direction *θ*_g_ and the consistency of head direction *ρ*:

2 $${\theta }_{{\rm{g}}}={\rm{atan}}2(\Sigma \sin ({\theta }_{{\rm{w}}}),\Sigma \cos ({\theta }_{{\rm{w}}}))$$

3 $${\rho }_{t}=\sqrt{{\left(\frac{\Sigma \cos ({\theta }_{{\rm{w}}})}{{N}_{{\rm{w}}}}\right)}^{2}+{\left(\frac{\Sigma \sin ({\theta }_{{\rm{w}}})}{{N}_{{\rm{w}}}}\right)}^{2}}$$

In equation (2), *θ*_g_ represents the goal direction associated with time point *t*, **θ**_w_ is a vector consisting of all head directions within the 15 s before and after time point *t* at which the fly was moving, and the atan2 function is the two-argument arctangent. As each head direction is treated as a unit vector we can simply convert each value of **θ**_w_ into Cartesian coordinates, calculate the sum of these values along each axis and take the arctangent to convert them back to polar coordinates to find the average angle the fly travelled at during that window. In equation (3), *ρ*_*t*_ represents the *ρ* value associated with time point *t*, and *N*_w_ is the number of data points over which *ρ* is calculated. Again, we first convert each *θ* value into Cartesian coordinates and find the average distance travelled along each axis before calculating *ρ*, so that *ρ* ranges between 0 and 1. Note that *ρ* = 1 would indicate that the fly maintained the same head direction for the entire window, while *ρ* = 0 would indicate that the fly uniformly sampled all possible head directions during the window. Figure 1g shows mean *ρ* and *θ* values from each trial, with radial length proportional to *ρ*.

### Path segmentation based on walking straightness

We observed that flies often walked in a straight line for an extended segment and then switched to a different apparent goal direction (*θ*_g_) to initiate a new segment (Extended Data Fig. 10). To infer the fly’s goal direction, we automatically divided each path into segments. We reasoned that a switch in *θ*_g_, would coincide with a dip in head direction consistency. Therefore, we looked for moments when *ρ* crossed a threshold value, and we broke the path into segments at those moments of threshold-crossing. The only exception was if *ρ* fell below threshold only very briefly (less than 0.5 s); here we did not count these as segment breaks, but lumped those time points together as part of a continuous segment with the preceding and following time points. We found that a threshold of *ρ* = 0.88 matched our commonsense notion of when a new segment should start, but varying the threshold value over a wide range (0.70–0.98) did not dramatically change the outcome of our segmentation process nor the resulting relationships between neural activity and behaviour.

We then calculated the average *θ* and *ρ* for each of these segments and used the mean *θ* value as the inferred goal head direction. For all analyses, segments were discarded if *ρ* was equal to 1, as this indicated the panels had not been initiated correctly and that the cue had remained in a single location for the duration of the trial. Segments were also discarded if the fly was inactive (that is, if the fly’s cumulative velocity was not above a threshold of 0.67 rad s^−1^ for at least 2 s). For population analyses shown in Figs. 1h, 2e–g and  3, all remaining segments were used regardless of *ρ*.

For the head direction tuning analysis shown in Fig. 4f,g we used a threshold of *ρ* = 0.7, and we only used data from segments where *ρ* ≥ 0.7. We lowered the threshold on *ρ* for this analysis because we needed to include a larger number of time points in the analysis, to improve the resolution for binning the activity of cells into groups defined by *θ*_p_ − *θ*_g_.

### Classifying jumps as ‘corrected, high *ρ*’ versus ‘uncorrected, low *ρ*’

To analyse cue jumps (Figs. 1f and  5e–h and Extended Data Figs. 3 and 7), we classified jumps as ‘corrected, high *ρ*’ or ‘uncorrected, low *ρ*’. Here we rejected jumps where the fly was essentially immobile in the epoch before the jump (meaning its cumulative speed did not exceed 0.67 rad s^−1^ for at least 1 s in the 15 s before the jump). For each jump, we measured the original mean head direction (*θ*) during the 15 s before the jump, and we judged jumps as ‘corrected’ if *θ* returned to within 30° of its original value for ±90° jumps, or within 60° for 180° jumps, in the 10 s after the jump. We classified a jump trial as ‘high *ρ*’ if the average *ρ* was equal to or greater than 0.88 as calculated over time points within the 15 s before the jump, where the fly’s cumulative speed was over 0.67 rad s^−1^ and ‘low *ρ*’ otherwise.

In principle, it is possible that the jumps we categorized as uncorrected might have happened (by chance) to produce a smaller absolute change in the distance between a fly’s head direction and a cell’s preferred head direction |Δ(*θ* − *θ*_p_)|, as compared to the jumps in the corrected category. If this sampling artefact existed, it could produce an overall smaller absolute change in membrane potential for uncorrected jumps, leading us to misinterpret this result. However, we found no difference in the variance of ∆(*θ* − *θ*_p_) or the mean value of |Δ(*θ* − *θ*_p_)| for uncorrected versus corrected jumps (Extended Data Fig. 7d).

### Computing average response to iontophoresis stimulation

For the plots shown in Fig. 2a,b and Extended Data Fig. 4, data from the ±10 s period around each ATP pulse were averaged within individual flies to get the fly-averaged response to the 100 ms, 200 ms, 300 ms and 500 ms pulses for the membrane potential, forward velocity, sideways velocity and rotational velocity (each condition had at least four repetitions per fly). We then calculated the grand mean and s.e.m. across all flies using these per-fly averages.

### Computing activity bump parameters

To track the amplitude and phase of PFL2 activity for analyses in Figs. 2c–g and 5j,k and Extended Data Figs. 3, 5 and 8, a sinusoid was fit independently to each time point of the *z*-scored Δ*F/F* activity across fan-shaped body and protocerebral bridge imaging trials:

4 $${\rm{PFL}}2\,\,{\rm{activity}}=a\times \sin (x-u)+c$$

Here, PFL2 activity is a vector of *z*-scored Δ*F/F* values at a single time point such that it has ten bins if from a protocerebral bridge trial or nine if from an fan-shaped body trial, corresponding to the number of ROIs specified for each region. Here, *u* sets the phase of the sinusoid, *c* is the vertical offset term, *a* represents the bump amplitude, and the position in brain space where the peak of the sinusoid is located defines the bump phase. A bump phase of +180° represents the rightmost position in the protocerebral bridge and fan-shaped body while a phase of −180° represents the leftmost position.

### Computing change in bump phase versus change in head direction

We calculated the relative changes in PFL2 bump phase and head direction in 1.5 s bins as shown in Fig. 2d. In each time window, we took the difference between start and end points for *θ* or bump phase. Positive differences represent a clockwise shift while a negative difference represents a counterclockwise shift. The relationship between changes in *θ* and changes in bump phase was strongest when a 200 ms lag was implemented, such that changes in bump phase lagged 200 ms behind changes in *θ*. The line of best fit for the relationship between the two variables was found with the polyfit and polyval functions. We then used the corrcoef function to find the correlation coefficients and *P* value of the relationship. We excluded indices where the adjusted *r*^2^ value of the sinusoidal fit for bump parameters was below 0.1 or the fly was not moving.

### Computing population activity as a function of behaviour

To determine the relationship between neural activity and various behavioural parameters (Figs. 2e–g and 3 and Extended Data Fig. 5) we binned conditioned data. Within each segment described above, indices with cumulative velocity less than 0.67 rad s^−1^ were removed, and head directions were recalculated to be relative to the inferred goal head directions, meaning that a negative value indicated that the fly was facing counterclockwise to its goal head direction, and a positive value indicated that the fly was facing clockwise to its goal head direction. The *z*-scored Δ*F/F* was then averaged within bins of 10° s^−1^ for rotational velocity, 1 mm s^−1^ for forward velocity, or 10° for head direction. For Fig. 3d,e and Extended Data Fig. 5, the sum or difference between right and left LAL activity was calculated per segment following binning. The mean and s.e.m. was then calculated across flies.

### Computing preferred head direction

To show preferred cell head direction in Fig. 4a, we divided the estimated baseline membrane voltage (see section ‘Patch-clamping’) into 20° bins, based on the fly’s head direction. We considered the preferred head direction to be the value with the maximum binned membrane potential. The amplitude of the preferred head direction was calculated by taking the difference between the maximum and minimum binned membrane potential values.

### Analysis of IPSPs

To detect IPSPs for analyses in Fig. 4d, we focused only on jump trials where the fly was essentially immobile, to avoid any confounds associated with the membrane potential fluctuations in these cells that are associated with movement transitions. Action potentials were first removed from the voltage trace by median filtering the membrane potential with a 25 ms window, then lightly smoothing (smoothdata function in MATLAB, window size 20 ms, using the loess method). We then calculated the derivative of the membrane potential (gradient function in MATLAB) and found local minima corresponding to periods of rapid decreases in membrane potential (findpeaks function in MATLAB, peak distance of 20 ms, threshold determined for each cell). We also generated a detrended version of the membrane potential by subtracting the median filtered membrane potential (500 ms window) and found local minima (findpeaks function in MATLAB, peak distance of 20 ms, threshold determined for each cell). We categorized IPSPs as indices where a negative peak was detected from the derivative of the membrane potential trace within 30 ms before a negative peak in the baseline corrected trace.

### Computing change in IPSP parameters as a function of the change in head direction

To examine changes in IPSP parameters as a function of change in head direction, ±5 s windows around cue jumps in which the fly did not move for the entire 10 s period were used (Fig. 4c). All jumps fitting this category were analysed for 20 of 27 neurons in this dataset; the remaining 7 neurons were not included, as there were no cue jumps around which the fly was stopped for the entire 10 s window around the jump.

Detected IPSP frequency was calculated for the 5 s before or after the cue jump. The change in frequency before jump versus after jump was then compared to change in head direction relative to the cell’s preferred head direction produced by the cue jump. This was determined by first finding the absolute angular difference between the head direction before the jump and the cell’s preferred head direction (see section ‘Computing preferred head direction’) and doing the same for the new head direction following the cue jump. Then the precue jump value was subtracted from the postcue jump value. This means that a negative value indicated that the head direction was closer to the cell’s preferred head direction following the jump while a positive value indicated that the distance between the head direction and the cell’s preferred head direction increased following the jump. The change in IPSP frequency was then plotted against the change in the distance from the cell’s preferred head direction for each jump. MATLAB’s polyfit and polyval functions were used to find the line of best fit for the relationship between the two variables, while the corrcoef function was used to find the correlation coefficients of the relationship. Additionally, we used an unbalanced two-factor ANOVA to determine the significance of the relationship between change in frequency and change in head direction compared to that with cell identity.

### Exploring interactions between goal head direction and single-cell head direction tuning curves

To explore how single-cell dynamics lead to the population level relationships between neural activity and behaviour, we first segmented electrophysiology data into groups of continuous data points based on their associated goal head directions and *ρ* values (see section ‘Trial segmentation based on walking straightness and inferred goal direction’). For each trial, the cell’s preferred heading was determined (see section ‘Computing preferred head direction’) and the difference between the preferred heading and goal was found (*θ*_g_ − *θ*_p_). Segments were assigned to 72° wide bins based on the *θ*_g_ − *θ*_p_ value and for each segment, the head direction tuning curve was recalculated for both firing rate and membrane potential, using the data points within the bin. For Fig. 4f,g, the minimum value of each tuning curve was calculated and subtracted from that tuning curve. Following the subtraction, the mean and s.e.m. values were calculated across all tuning curves within each *θ*_g_ − *θ*_p_ bin. For Extended Data Fig. 6, the only difference is that the minimum value of the tuning curves was not subtracted.

### Determining the temporal relationship between neural activity and behaviour

The figures shown in Fig. 5h were created using the same method as previous jump analyses (see section ‘Classifying jumps as “corrected, high *ρ*” versus “uncorrected, low *ρ*”’) but pooling data across the PFL2 and PFL3 corrected jumps. For Extended Data Fig. 7e, jumps were categorized as corrected as done previously, except jumps were deemed corrected if within 4 s following the cue jump, the cue was returned to within 40° for ±90° jumps, or within 75° for 180° jumps. This was done to select for jumps where the fly initiated a behavioural response quite rapidly following the cue jump, as behavioural response times varied across and within flies. For each corrected jump, the mean membrane potential was calculated from data in the 4 s preceding the cue jump and subtracted from the membrane potential in the 4 s following the cue jump, in order to focus on the change in membrane potential. Pearson’s linear correlation coefficient was then found between the absolute change in membrane potential from the 4 s following the jump and the lagged copies of the rotational speed over the same time window using MATLAB’s corr function. The mean and s.e.m. for each lag (stepped by 0.01 s with a maximum and minimum lag of ±1 s) across all individual correlations was then calculated.

### Examining single-cell responses around cue jumps

For Fig. 5e–g and Extended Data Fig. 7a–d, jumps were categorized as either corrected or uncorrected as described previously (see section ‘Classifying jumps as “corrected, high *ρ*” versus “uncorrected, low *ρ*”’). For each jump, the difference between the mean membrane potentials calculated over the 1 s before and following each jump was found, and the distribution of these values is shown for both categories in Fig. 5f,g. A two sample Brown–Forsythe test was used to determine whether the variance of membrane potential changes was significantly different between the two categories.

### Examining the relationship between PFL2 activity and consistency of head direction

For Fig. 5j,k and Extended Data Fig. 8, we binned data from each ROI (protocerebral bridge glomerulus) individually across the entire non-segmented trial to obtain the average response of each glomerulus across different values of (*θ* − *θ*_g_). Here we inferred *θ*_g_ from neural activity rather than behaviour, because we wanted to include epochs with low *ρ*, and it is difficult to infer *θ*_g_ from the fly’s behaviour when *ρ* is low. To infer *θ*_g_ from neural activity, we grouped PFL2 bump amplitude data points by *θ* in 5˚ bins, and we calculated the difference in bump amplitude between pairs of bins 180˚ apart. Our model predicts that the absolute bump amplitude difference should be largest between the bins representing the goal and anti-goal, and so we searched for the pair of opposing bins with the largest difference in bump amplitude, and we took *θ*_g_ as the value of *θ* corresponding to the bin with the smaller bump amplitude. For Fig. 5k, we plotted the largest bump amplitude difference against the trial’s average *ρ* value, as calculated over the entire trial. For the individual brain space plots shown in Fig. 5i,j and Extended Data Fig. 8, we used this *θ*_g_ value to calculate the directional error (*θ* − *θ*_g_) and we binned the *z*-scored Δ*F/F* data points from each individual ROI into 90˚ bins based on their associated directional error value. We then plotted the *z*-scored Δ*F/F* within each directional error bin against neural space (ROI identity), with the rightmost glomeruli represented by an angular position of +180° and the leftmost by −180°.

Note that this analysis assumes that *θ*_g_ does not change very much over the course of a trial. If *θ*_g_ did change dramatically, this would result in a lower *ρ* value for the trial and possibly also a reduced bump amplitude range value, despite the fly potentially being in a state of high goal fixation strength for the entire trial. Flies that switched between periods of very strong and weak goal fixation would be expected to result in a similar potential mismatch between *ρ* and bump amplitude range. Therefore, the limitations of the analysis in Fig. 5k should, if anything, reduce our ability to detect a relationship between PFL2 activity and behaviour.

### Neurotransmitter predictions

There are 12 complete PFL2 cells, 13 complete PFL3 cells and one nearly complete DNa03 cell in the hemibrain connectome, with over 100 presynapses associated with each of these cells. Although the axon terminal of DNa03 is not present in the hemibrain dataset, DNa03 makes many output synapses in the brain, so there are still many EM images of the presynaptic sites within this cell. A recent algorithm^50,51^ automatically infers transmitter identification from electron micrographs in the hemibrain dataset, and it predicts that, of these, 12 of 12 PFL2 neurons are cholinergic, 13 of 13 PFL3 neurons are cholinergic and 1 of 1 DNa03 neuron is cholinergic. This algorithm predicts transmitters on a per-synapse basis, with an error rate that varies with cell and transmitter type. For PFL2 and PFL3 neurons, 74% of high-confidence presynapses (confidence score greater than or equal to 0.5) are predicted as cholinergic; the second most commonly predicted transmitter is glutamate (11%). For DNa03, 85.2 % of high-confidence presynapses are predicted as cholinergic; the second most commonly predicted neurotransmitter is glutamate (5.6%). This algorithm used 3,094 hemibrain neurons in its ground-truth data to train the model and included ground truth neurons identified as cholinergic using light microscopy pipelines and antibody staining or RNA sequencing. Among this ground-truth population, 73% of presynapses are correctly predicted as cholinergic. All synapse predictions are available from ref. ^51^.

### Connectome analyses

Cell connectivity data was obtained from the hemibrain connectome at https://neuprint.janelia.org/ and analysis of this data was performed using the neuprintr natverse 1.1 software package^52^ available at https://natverse.org/.

### Network model

Our model shares features with several other recent models of central complex steering control^4,5,11–13^. These studies, in turn, built upon the existing idea that vectors should be represented as sinusoidal spatial patterns of neural activity, so that vector addition can be implemented via the addition of sinusoids^9,15,16,53,54^. Webb and colleagues extended this idea to an explicit notion of how rotational velocity commands might be generated via vector addition, by using right–left shifted basis vectors^9^. While our model incorporates these previous insights, it also takes advantage of new information from the automatic assignment of neurotransmitters^50^, as well as our neurophysiological experiments. For these reasons, it differs from previous models in a few important ways, as noted below. Most notably, our model shows how this network can adaptively control steering gain based on the magnitude of directional error (via PFL2 cells). Previous studies did not mention PFL2 cells, or else proposed that they have a non-steering-related role (as putative positive regulators of forward speed^5,13^). In contrast, our model gives these cells a strong influence over steering, and it shows how they can prevent oscillations in the steering system by boosting steering only when error is high, while throttling down steering when error is low.

In broad terms, the aim of the model is to understand how steering signals arise from the head direction system. We take the steering signal as the right–left difference in the activity of DNa02 descending neurons, because these neurons have been shown to predict and influence steering^4^:

5 $${\rm{d}}\theta /{\rm{d}}t\propto {\rm{DNa02R\; -\; DNa02L}}+{\epsilon }$$

where *θ* is head direction and *ε* is a random term that accounts for neural noise and the influence of unmodeled circuits (that is, the influence of other brain regions that affect steering and other descending pathways^4,55,41^). Here, (${\rm{d}}\theta /{\rm{d}}t$ > 0) denotes rightward (clockwise) steering.

DNa02 receives direct input from central complex output neurons (PFL3 cells), as well as indirect input from PFL2 and PFL3 cells via DNa03. We model the activity of each DNa02 cell by taking the weighted sum of its synaptic inputs and passing this through a nonlinearity:

6 $$\begin{array}{c}{\rm{DNa}}02{\rm{R}}=f(\Sigma {W}_{{\rm{DNa}}02{\rm{R}},{\rm{PFL}}3{{\rm{R}}}_{j}}\times {\rm{PFL}}3{{\rm{R}}}_{j}+\Sigma {W}_{{\rm{DNa}}02{\rm{R}},{\rm{DNa}}03{\rm{R}}}\times {\rm{DNa}}03{\rm{R}})\\ \\ {\rm{DNa}}02{\rm{L}}=f(\Sigma {W}_{{\rm{DNa}}02{\rm{L}},{\rm{PFL}}3{{\rm{L}}}_{j}}\times {\rm{PFL}}3{{\rm{L}}}_{j}+\Sigma {W}_{{\rm{DNa}}02{\rm{L}},{\rm{DNa}}03{\rm{L}}}\times {\rm{DNa}}03{\rm{L}})\end{array}$$

where $W$ denotes an array of synaptic weights and $f$ represents a nonlinear activation function (see below). We define PFL3R cells as the members of the PFL3 cell class that project their axons to the right hemisphere; PFL3L cells are the members of the PFL3 cell class that project their axons to the left hemisphere. This differs from some previous work where PFL3 cells were divided according to dendritic location rather than their axonal projection^5^.

We model the activity of each DNa03 cell by taking the weighted sum of its synaptic inputs and passing this sum through the same type of nonlinearity. Here the relevant inputs to each DNa03 cell are from PFL3 cells and PFL2 cells. Each PFL2 axon projects bilaterally to both right and left brain hemispheres, and we model these connections as right–left symmetric, because we do not find any systematic asymmetry in connectome data; thus we use the same weights for PFL2 connections onto DNa03R and DNa03L:

7 $$\begin{array}{c}{\rm{DNa}}03{\rm{R}}=f(\Sigma {W}_{{\rm{DNa}}03{\rm{R}},{\rm{PFL}}3{{\rm{R}}}_{j}}\times {\rm{PFL}}3{{\rm{R}}}_{j}+\Sigma {W}_{{\rm{DNa}}03,{\rm{PFL}}{2}_{j}}\times {\rm{PFL}}{2}_{j})\\ \\ {\rm{DNa}}03{\rm{L}}=f(\Sigma {W}_{{\rm{DNa}}03{\rm{L}},{\rm{PFL}}3{{\rm{L}}}_{j}}\times {\rm{PFL}}3{{\rm{L}}}_{j}+\Sigma {W}_{{\rm{DNa}}03,{\rm{PFL}}{2}_{j}}\times {\rm{PFL}}{2}_{j})\end{array}$$

We then combine equations (5)–(7) to obtain an expression that predicts steering as a function of PFL2 and PFL3 activity. Here we assume that DNa03 output is anatomically symmetric in the right and left hemispheres. For compactness, we notate weight arrays using the abbreviations D2 (DNa02), D3 (DNa03) P2 (PFL2) and P3 (PFL3):

8 $$\begin{array}{l}{\rm{d}}\theta /{\rm{d}}t\propto {\rm{DNa}}02{\rm{R}}-{\rm{DNa}}02{\rm{L}}+{\epsilon }\\ =\,f(\Sigma {W}_{{\rm{D2R}},{\rm{P}}3{{\rm{R}}}_{j}}\times {{\rm{P3R}}}_{j}+\Sigma {W}_{{\rm{D2,D3}}}\times {\rm{D3R}})\\ \,-f(\Sigma {W}_{{{\rm{D2L,P3L}}}_{j}}\times {{\rm{P3L}}}_{j}+\Sigma {W}_{{\rm{D2,D3}}}\times {\rm{D3L}})+{\epsilon }\\ =\,f(\Sigma {W}_{{{\rm{D2R,P3R}}}_{j}}\times {{\rm{P3R}}}_{j}+\Sigma {W}_{{\rm{D2,D3}}}\times f(\Sigma {W}_{{{\rm{D3R,P3R}}}_{j}}\times {{\rm{P3R}}}_{j}\\ \,+\Sigma {W}_{{{\rm{D3,PFL2}}}_{j}}\times {{\rm{PFL2}}}_{j}))\\ \,-f(\Sigma {W}_{{{\rm{D2L,P3L}}}_{j}}\times {{\rm{P3L}}}_{j}+\Sigma {W}_{{\rm{D2,D3}}}\times f(\Sigma {W}_{{{\rm{D3L,P3L}}}_{j}}\times {{\rm{P3L}}}_{j}\\ \,+\Sigma {W}_{{{\rm{D3,PFL2}}}_{j}}\times {{\rm{PFL2}}}_{j}))+{\epsilon }\end{array}$$

If the activation function $f$ is linear, the PFL2 terms will cancel out and PFL2 cells will have no effect on steering; therefore, we require $f$ to be nonlinear, at least for DNa03 cells. Below we will see that $f$ must also be nonlinear for PFL3 cells. For consistency, we give $f$ the same form for all cells in the model (see below). If $f$ is an expansive nonlinearity and if PFL2 cells are excitatory (as inferred from neurotransmitter predictions, see above), then PFL2 cells will increase the gain of steering commands, because they push DNa03 cells up into the steeper part of the nonlinearity.

We specify the weight array $W$ for each connection type based on data from the hemibrain 1.2.1 (ref. ^5^) connectome, following the heuristic that functional weights are roughly proportional to the number of synaptic contacts per unitary connection^42,45^. Connectome data imply that PFL3 → DNa03 connections are approximately equal in strength to PFL3 → DNa02 connections, on average; all these weights are set to 1 in our model. Meanwhile, connectome data imply that PFL2 → DNa03 connections are approximately 4-fold stronger than PFL3 → DNa02 and PFL3 → DNa03 connections, on average; therefore, we set PFL2 → DNa03 weights equal to 4. Finally, connectome data imply that DNa03 → DNa02 connections are approximately 12-fold stronger than PFL3 → DNa02 and PFL3 → DNa03 connections; therefore, we set DNa03 → DNa02 connections to 12. We verified that our conclusions were not altered if we chose somewhat different scaling factors for these connections. Within each weight array $W$, we set all entries to the same value; in other words, all connections of the same type were given the same weight. All weights were positive, because all the presynaptic cells are cholinergic and thus excitatory (see section ‘Neurotransmitter predictions’). Some previous studies assumed that PFL3 cells are inhibitory^5,11^, which produces different model behaviour, because it aligns the system’s stable fixed point with the point of maximum PFL2 activity (not the minimum of PFL2 activity), resulting in more oscillatory steering around the goal.

Our model contains 1,000 PFL2 units, 1,000 PFL3R units, 1,000 PFL3L units and 1,000 goal cell units. We chose to use a large number of units for these cell types, so that model output resembles a quasi-continuous function over neural space, because this makes it easier to see how spatial patterns of ensemble neural activity might resemble a sinusoidal function. In reality, however, there are only 12 PFL2 cells, 12 PFL3R cells and 12 PFL3L cells in the brain, according to the hemibrain 1.2.1 (ref. ^5^) connectome, so activity in the brain is actually more discretized than in our model. We verified that discretizing neural activity to match these numbers does not alter our conclusions.

In our model, the activity of each PFL cell depends on both head direction and goal direction. Δ7 cells provide most of the head direction input to PFL2 and PFL3 cells^5^. Available data indicate that there are two complete linearized topographic maps of head direction in Δ7 cells, positioned side-by-side and formatted as two cycles of a sinusoidal function over neural space^5,24,47,56^. The spatial phase of the Δ7 activity pattern should have an arbitrary offset (*θ*_0_) relative to the fly’s head direction, with different values of *θ*_0_ in different individuals and at different times in the same individual, because this is true of EPG cells, which provide head direction input to Δ7 (ref. ^19^). We define the offset *θ*_0_ as the angular position of the EPG bump at a head direction of 0°. For simplicity, we lump the contributions of EPG output and Δ7 cells, and we treat their lumped contributions as a sinusoidal function over neural space. Specifically, we model their lumped output as cos(*θ* − *θ*_0_ − **h**), where **h** is a vector with 1,000 entries that uniformly tile the full 360° of angular space, representing the preferred head directions of 1,000 units. As the fly rotates rightward (clockwise), the sinusoidal pattern of neural activity moves leftward across the protocerebral bridge^47,56^.

We define PFL3 cells as R or L depending on whether they project to right or left descending neurons, respectively. The head direction maps in PFL3 cells are shifted ±67.5° relative to the map in Δ7 cells, according to hemibrain connectome data^41^ (not ±90° as reported previously^5,12,13^). Therefore, we model the head direction input to PFL3R cells as cos(*θ* − *θ*_0_ − **h** + 67.5°), and we model the head direction input to PFL3L cells as cos(*θ* − *θ*_0_ − **h** − 67.5°). Meanwhile, PFL2 cells sample one full head direction map from the middle section of the protocerebral bridge. Therefore, their head direction map is offset by 180°, relative to the map in Δ7 cells. Thus, we model the head direction input to PFL2 cells as cos(*θ* − *θ*_0_ − **h** + 180°).

We model the neural representation of the goal direction (*θ*_g_) as another sinusoidal pattern over neural space, which is reasonable, because the goal direction can be thought of as just a special head direction, and head direction is represented as a sinusoid. Because PFL2, PFL3R and PFL3L cells receive almost identical inputs in the fan-shaped body, we assume the goal input is the same in the PFL2, PFL3R and PFL3L populations. The output of goal cells is modelled as *A* × cos(*θ*_g_ − *θ*_0_ − **h**); note that if there is a shift in the offset of the head direction system (*θ*_0_), the goal representation will shift accordingly. As the goal direction rotates rightward (clockwise), the peak of activity in goal cells will move leftward across the fan-shaped body. We use *A* = 1 in our model implementations, so that the amplitude of the goal signal is equal to the amplitude of the head direction signal, but some of our results can be potentially explained by a mechanism that modulates *A* (Extended Data Fig. 8).

To obtain PFL activity levels, we sum head direction inputs and goal inputs. We then rescale this sum according to a scaling factor *S*. Finally, we pass the result through a nonlinear activation function $f$:

9 $$\begin{array}{l}\,\,{\rm{PFL}}2\,=\,f(S\times (\cos (\theta -{\theta }_{0}-{\bf{h}}+{180}^{^\circ })+A\times \,\cos ({\theta }_{g}-{\theta }_{0}-{\bf{h}})))\\ {\rm{PFL}}3{\rm{R}}\,=\,f(S\times (\cos (\theta -{\theta }_{0}-{\bf{h}}+{67.5}^{^\circ })+A\times \,\cos ({\theta }_{g}-{\theta }_{0}-{\bf{h}})))\\ {\rm{PFL}}3{\rm{L}}\,=\,f(S\times (\cos (\theta -{\theta }_{0}-{\bf{h}}-{67.5}^{^\circ })+A\times \,\cos ({\theta }_{g}-{\theta }_{0}-{\bf{h}})))\end{array}$$

Note that the activation function $f$ must be nonlinear or else the goal input will have no influence on the right–left difference in PFL3 activity (ΣPFL3R − ΣPFL3L). We use *S* = 1 by default, except in Figs. 5c,d and 5j,k, where we investigate the effect of lowering *S*.

For simplicity, we use the same nonlinear activation function $f$ for all units in this model (meaning all PFL2, PFL3, DNa03 and DNa02 cells). Specifically, we use an exponential linear unit or ELU. We chose an ELU because it is biologically highly plausible (as a ‘soft’ expansive nonlinearity^57^) and it is a good fit to our data. The input to the ELU is an array *M* that represents the weighted sum of the inputs to each cell, over its lifetime, for all values of head direction (*θ*), goal direction (*θ*_g_), scaling parameter (*S*) and cell index (*j*). We rescale *M* so that min(*M*) = −1 and max(*M*) = 1. Then, we apply the function

10 $$\begin{array}{c}{\rm{ELU}}(M)=M\,{\rm{for}}\,M\ge 0\\ \\ {\rm{ELU}}(M)={{\rm{e}}}^{M}-1\,{\rm{for}}\,M < 0\end{array}$$

before finally rescaling the resulting array ${\rm{ELU}}(M)$ so that it ranges from 0 to 1. These rescaling procedures are motivated by the idea that a neuron’s inputs are adjusted (over development and/or evolution) to fit into some standard dynamic range dictated by the biophysical properties of a typical neuron; rescaling in this way is useful because it ensures that every cell type has a similar overall level of activity, and every cell has an activation function with the same shape. Note that from the perspective of a single PFL cell, the goal input is a fixed value that does not change as head direction changes, and when this goal signal becomes more positive (again, from the perspective of a single PFL cell), it pushes the cell’s activity up to a steeper part of the nonlinear function $f$, effectively amplifying the cell’s head direction tuning. This aspect of the model captures our experimental observation that head direction tuning is stronger in some cells than in other cells, in a way that depends systematically on the distance between the cell’s preferred head direction (*θ*_p_) and the goal direction (*θ*_g_). Notably, this observation emerges only at the level of spike rate, not membrane potential (Fig. 4f,g), and this implies that the nonlinearity $f$ is largely due to the voltage-gated conductances that transform total synaptic input into spike rate. We verified that the basic conclusions of our model are unchanged if we substitute different nonlinear activation functions (sigmoid or ReLU rather than ELU); other published models have assumed a multiplicative^12^ or divisive nonlinearity^13^.

To model steering behaviour over time (Fig. 5d), we closed the loop on the brain’s feedback control system for steering: we took the fly’s predicted rotational velocity (${\rm{d}}\theta /{\rm{d}}t$) at each time point, and we fed it back into the head direction representation at the next time point, in order to compute updated PFL2 and PFL3 activity. The simulation was updated at a frequency of 10 Hz, and Fig. 5d shows 10 s of simulated time. We arbitrarily took 0° as the goal direction, so directional error is equal to *θ*. We drew the random steering component *ε* (equation (5)) from a Gaussian distribution, then we low-pass filtered *ε*(*t*) at 2 Hz, before rescaling *ε*(*t*) to enforce a standard deviation of 10°. This was done for different values of *S*, using the same frozen noise sample *ε*(*t*) in each case. In Fig. 5k, we used many independent random samples of *ε*(*t*), each simulation run included 100 s of simulated time, and we swept through many values of *S*, computing PFL2 bump amplitude and the consistency of head direction (*p*) for each run, with *p* = (one-circular variance(*θ*)). Model code was written and implemented in Python v.3.9.5.

### Reporting summary

Further information on research design is available in the Nature Portfolio Reporting Summary linked to this article.

## Online content

Any methods, additional references, Nature Portfolio reporting summaries, source data, extended data, supplementary information, acknowledgements, peer review information; details of author contributions and competing interests; and statements of data and code availability are available at 10.1038/s41586-024-07039-2.

## Supplementary information

Reporting Summary

## Data Availability

The datasets generated and/or analysed during the current study are available from the corresponding author on reasonable request.

## Code availability

Code for data analysis and model implementation is available at https://github.com/wilson-lab/WesteindeWilson_AnalysisCode.
