# Author Correction: Transforming a head direction signal into a goal-oriented steering command

**DOI:** 10.1038/s41586-024-08245-8

**Published:** 2025-03-11

**Authors:** Elena A. Westeinde, Emily Kellogg, Paul M. Dawson, Jenny Lu, Lydia Hamburg, Benjamin Midler, Shaul Druckmann, Rachel I. Wilson

**Affiliations:** 1 Department of Neurobiology, Harvard Medical School, Boston, MA USA; 2 Department of Neurobiology, Stanford University School of Medicine, Stanford, CA USA; 3 Wu Tsai Neurosciences Institute, Stanford University, Stanford, CA USA

**Keywords:** Network models, Navigation

Correction to: *Nature* 10.1038/s41586-024-07039-2 Published online 7 February 2024

In the version of the article initially published, in the “Flies” and “Origins of transgenic stocks” sections of the Methods, one genotype listed, +;P{VT033284-p65AD}attP40; P{y[+t7.7];P{VT007338-Gal4DBD}attP2, was incorrect and has now been amended to +;P{VT007338-p65ADZp}attP40;P{y[+t7.7] w[+mC]=VT044709-GAL4.DBD}attP2. Additionally, the heading of Extended Data Fig. 2c was “PFL2 split-Gal4 line: *VT033284AD;VT007338DBD”* and has now been amended to “PFL2 split-Gal4 line: *VT007338AD;VT044709DBD*”. These corrections have been made to the HTML and PDF versions of the article.

