# Supplementary material for: Transforming a head direction signal into a goal-oriented steering command
Source: Nature. 2024 Feb 7;626(8000):819–26. doi: 10.1038/s41586-024-07039-2 (PMC10881397; doi:10.1038/s41586-024-07039-2)
Supplement: Supplementary file 1 — Reporting Summary [file 41586_2024_7039_MOESM1_ESM.pdf]

nature portfolio

Corresponding author(s): Rachel Wilson

Last updated by author(s): 2023/11/13

Reporting Summary

Nature Portfolio wishes to improve the reproducibility of the work that we publish. This form provides structure for consistency and transparency in reporting. For further information on Nature Portfolio policies, see our Editorial Policies and the Editorial Policy Checklist.

Statistics

For all statistical analyses, confirm that the following items are present in the figure legend, table legend, main text, or Methods section.

n/a	Confirmed	
☐	☒	The exact sample size (*n*) for each experimental group/condition, given as a discrete number and unit of measurement
☐	☒	A statement on whether measurements were taken from distinct samples or whether the same sample was measured repeatedly
☐	☒	The statistical test(s) used AND whether they are one- or two-sided<br>*Only common tests should be described solely by name; describe more complex techniques in the Methods section.*
☐	☒	A description of all covariates tested
☐	☒	A description of any assumptions or corrections, such as tests of normality and adjustment for multiple comparisons
☐	☒	A full description of the statistical parameters including central tendency (e.g. means) or other basic estimates (e.g. regression coefficient) AND variation (e.g. standard deviation) or associated estimates of uncertainty (e.g. confidence intervals)
☐	☒	For null hypothesis testing, the test statistic (e.g. *F*, *t*, *r*) with confidence intervals, effect sizes, degrees of freedom and *P* value noted<br>*Give P values as exact values whenever suitable.*
☒	☐	For Bayesian analysis, information on the choice of priors and Markov chain Monte Carlo settings
☒	☐	For hierarchical and complex designs, identification of the appropriate level for tests and full reporting of outcomes
☐	☒	Estimates of effect sizes (e.g. Cohen's *d*, Pearson's *r*), indicating how they were calculated

Our web collection on statistics for biologists contains articles on many of the points above.

Software and code

Policy information about availability of computer code

Data collection	MATLAB 2019b, R2021a, ScanImage 2021, FicTrac v2.1 (https://github.com/rjdmoore/fictrac), neuprint (https://neuprint.janelia.org/), NeuprintR 1.1 (https://github.com/natverse/neuprintr) and natverse 1.1 (https://github.com/natverse/natverse)
Data analysis	Motion correction of calcium imaging data was performed using NoRMCorre (https://github.com/flatironinstitute/NoRMCorre). Ball tracking was performed using FicTrac v2.1. Computational modeling and analysis of calcium imaging, behavior, and electrophysiology data was performed using custom code written in MATLAB (2019b, R2021a) and Python 3.9.5. Code will be deposited in a public repository (e.g., github or zenodo) at the time of publication.

For manuscripts utilizing custom algorithms or software that are central to the research but not yet described in published literature, software must be made available to editors and reviewers. We strongly encourage code deposition in a community repository (e.g. GitHub). See the Nature Portfolio guidelines for submitting code & software for further information.

Data

Policy information about availability of data

All manuscripts must include a data availability statement. This statement should provide the following information, where applicable:

- Accession codes, unique identifiers, or web links for publicly available datasets
- A description of any restrictions on data availability
- For clinical datasets or third party data, please ensure that the statement adheres to our policy

The hemibrain v1.2.1 connectome data is available via a publicly accessible website, https://neuprint.janelia.org (also accessible via https://doi.org/10.25378/janelia.11676099.v2). The datasets generated during the current study are available from the corresponding author on reasonable request.

NOTE TO EDITOR: the datasets we collected are extremely large and have complex and unique metadata structures associated with them, making deposition of the data in a public repository impractical but are available from the corresponding author on reasonable request.

Human research participants

Policy information about studies involving human research participants and Sex and Gender in Research.

Reporting on sex and gender	n/a
Population characteristics	n/a
Recruitment	n/a
Ethics oversight	n/a

Note that full information on the approval of the study protocol must also be provided in the manuscript.

Field-specific reporting

Please select the one below that is the best fit for your research. If you are not sure, read the appropriate sections before making your selection.

☒ Life sciences ☐ Behavioural & social sciences ☐ Ecological, evolutionary & environmental sciences

For a reference copy of the document with all sections, see nature.com/documents/nr-reporting-summary-flat.pdf

Life sciences study design

All studies must disclose on these points even when the disclosure is negative.

Sample size	All sample sizes were chosen based on conventions in our field for standard sample sizes. These sample sizes are conventionally determined on the basis of the expected magnitude of animal-to-animal variability, given published results and pilot data. Statistical analyses were not performed until data collection was completed. No formal power calculations were performed due to the expected variability and exploratory nature of the dataset.
Data exclusions	We did not exclude any flies from the calcium imaging, iontophoresis, or electrophysiology datasets. Trial segments were excluded from analyses shown in Fig 1h, 2e-g, 3, 4d, and Extended Data Fig 5 if the fly only sampled a single heading during the entire segment, as this indicated that the visual arena did not initialize properly at the beginning of the segment (a technical problem that occurred rarely but in a few trials) . Trial segments were also excluded if the fly's total velocity was not above a set threshold for at least 2 seconds as this provided an insufficient time window to measure the fly's likely goal. In Fig 4f,g, and Extended Data Fig 6a data was excluded as described in the methods as required by the definition of the analysis to focus on segments with high associated values of rho. Rho threshold values were set empirically but we confirmed that small changes in this threshold did not change our conclusions, as described in the methods.
Replication	For all experiments, results were replicated in different individual flies across each dataset, the number of replicates performed are described in the figure legends. We did not omit any replicates on the basis of the experimental result. A few trials were excluded due to factors that prevented us from analyzing the data; all these cases of data exclusion are noted explicitly above and in the Online Methods
Randomization	For PFL2 activation experiments (Fig. 2, Extended data Fig. 5) flies were grouped for analysis based on genotype. Beyond these cases, flies were not assigned to treatment groups. For all other experiments allocation of data into different categories is described in the associated methods sections.
Blinding	The experimenter was not blind to genotype in this study. This is because the different genotypes in the study were used to target a genetically encoded fluorescent indicator to different cell types, and so the genotype of the flies was obvious during the course of the experiments, based on the observed pattern of fluorescence.

Reporting for specific materials, systems and methods

We require information from authors about some types of materials, experimental systems and methods used in many studies. Here, indicate whether each material, system or method listed is relevant to your study. If you are not sure if a list item applies to your research, read the appropriate section before selecting a response.

Materials & experimental systems

n/a	Involved in the study
☐	☒ Antibodies
☒	☐ Eukaryotic cell lines
☒	☐ Palaeontology and archaeology
☐	☒ Animals and other organisms
☒	☐ Clinical data
☒	☐ Dual use research of concern

Methods

n/a	Involved in the study
☒	☐ ChIP-seq
☒	☐ Flow cytometry
☒	☐ MRI-based neuroimaging

Antibodies

Antibodies used

chicken anti-GFP (1:1,000, Abcam, # ab13970), mouse anti-Bruchpilot (1:30, Developmental Studies Hybridoma Bank, nc82), Alexa Fluor 488 goat anti-chicken (1:250, Invitrogen, #A11039), Alexa Fluor 633 goat anti-mouse (1:250, Invitrogen, #A21050), streptavidin::Alexa Fluor 568 (1:1000, Invitrogen, #S11226) , rat anti-Flag (1:200, Novus Biologicals, #NBP1-06712B), rabbit anti-HA (1:300, Cell Signal Technologies, #NBP106712B), Alexa Fluor 488 goat anti-rabbit (1:250, Invitrogen, #A11039), ATTO 647 goat anti-rat (1:400, Rockland, #612-156-120), Alexa Fluor 405 goat anti-mouse (1:500, Invitrogen, #A31553), DyLight 550 mouse anti-V5 (1:500, Bio-Rad, #MCA1360D550GA)

Validation

The anti-GFP antibody (Abcam) is the standard antibody used in the field for labeling Green Fluorescent Protein (GFP) in Drosophila, note that this protein is not endogenously expressed in the Drosophila genome. Manufacturer's datasheet confirm that this anti-GFP antibody has been validated using western blot and immunohistochemistry to have specificity for Green Fluorescent Protein. Manufacturer also confirms the use of this antibody for immunolabeling of GFP in Drosophila across 3182 peer-reviewed manuscripts (e.g. Sykes et al. 2005 PMID: 16122730). The antibruchpilot antibody (nc82, DSHB) is a standard in the field as a background stain that labels presynaptic active zones to provide neuropil labeling for analysis of anatomy. This antibody was originally validated for use in Drosophila to label presynaptic active zones using immunohistochemistry and to be specific to Bruchpilot protein (Wagh et al. 2006). The secondary antibody we used to label GFP expressing cells (Alexa Fluor 488 goat anti-chicken) was verified by us to target only those cells which express live GFP fluorescence. The secondary antibody used for background (neuropil) staining (Alexa Fluor 488 goat anti-chicken, Alexa Fluor goat anti-mouse 633) was verified by us to reproduce the known patterns of neuropil borders (nC82 immunoreactivity) in published atlases (VirtualFlyBrain.org). The streptavidin::Alexa Fluor 568 for visualizing cell fills was verified by us to only label a single cell in a given brain, the one filled with neurobiotin citrate during the experiment.

Antibodies used for MCFO immunostaining (rat anti-FLAG, rabbit anti-HA, DyLight 550 mouse anti-V5, AlexaFluor 488 goat anti-rabbit, ATTO 647 goat anti-rat) are validated in Drosophila melanogaster for this application in Nern et al., 2015. These antibodies have also each been validated prior to Nern et al:
rat anti-FLAG: Manufacturer notes confirms that rat anti-FLAG (Cat#: NBP1-06712B) has also been validated as FLAG-Tag specific in Drosophila (PMID: 26573957). Rabbit anti-HA: Manufacturer confirmed rabbit anti-HA antibody has Epitope tag specificity using western blot and immunohistochemical analysis comparing untransfected with HA-tag transfected COS cells (https://www.cellsignal.com/products/primary-antibodies/ha-tag-c29f4-rabbit-mab/3724#validation-data). DyLight 550-conjugated mouse anti-V5: Manufacturer notes confirm that the DyLight 550-conjugated-Mouse anti V5-Tag, clone SV5-Pk1 recognizes the sequence, IPNPLLGLD, present on the P/V proteins of the paramyxovirus, SV5 (Dunn et al.1999) and can be used to detect recombinant proteins labeled with this V5-tag (Randall et al.1993 and Zhao et al. 2005).

Animals and other research organisms

Policy information about studies involving animals; ARRIVE guidelines recommended for reporting animal research, and Sex and Gender in Research

Laboratory animals

We used female Drosophila melanogaster flies for all experiments. Newly eclosed flies were collected ~16-24 hrs (electrophysiology) or 1-4 days (imaging) before the experiment.

The following stocks were obtained from Well Genetics: w[1118];P{VT007338-p65ADZp}attP40/CyO;+ (A/SWG9178), w[1118];P{VT033284-p65AD}attP40/CyO;+ (A/SWG8077).

The following stocks were obtained from the Bloomington Drosophila Stock Center (BDSC) and published previously: P{y[+t7.7]w[+mC]=VT044709-GAL4.DBD}attP2 (BDSC_75555), P{y[+t7.7]w[+mC]=p65.AD.Uw}attP40; P{y[+t7.7] w[+mC]=GAL4.DBD.Uw}attP2 (BDSC_79603), P{w[+mC]=UAS-Rnor\P2rx2.L}4/CyO (BDSC_91223), w[1118]; PBac{y[+mDint2] w[+mC]=20XUAS-IVS-jGCaMP7b}VK00005. w+;20XUAS-cyRFP {VK00037};+ was obtained in house and P{20XUAS-IVS-mCD8::GFP}attP40 was a gift from Gerry Rubin and has been published previously

We constructed a split-Gal4 line to target PFL2 neurons, w+ ;P{VT033284-p65AD}attP40; P{y[+t7.7];P{VT007338-Gal4DBD}attP2. We

validated the expression of this line using immunohistochemical anti-GFP staining, and also using Multi-Color-Flip-Out to visualize single-cell morphologies. We also constructed a split-Gal4 line that targets PFL2 & PFL3 neurons in the lateral accessory lobes, w+;P{VT033284-p65AD}attP40;P{y[+t7.7] w[+mC]=VT044709-GAL4.DBD}attP2. We validated the expression of this line using immunohistochemical anti-GFP staining, and also using Multi-Color-Flip-Out.
(MCFO) to visualize single-cell morphologies.

Wild animals	No wild animals were used in this study.
Reporting on sex	All animals used in this study were female, due to the experimental difficulty presented by the use of male flies (which are smaller).
Field-collected samples	No field samples were collected for this study.
Ethics oversight	No ethical approval was required because experiments were performed on Drosophila melanogaster.

Note that full information on the approval of the study protocol must also be provided in the manuscript.
